# Transcriptome analysis at four developmental stages of grape berry (*Vitis vinifera* cv. Shiraz) provides insights into regulated and coordinated gene expression

**DOI:** 10.1186/1471-2164-13-691

**Published:** 2012-12-11

**Authors:** Crystal Sweetman, Darren CJ Wong, Christopher M Ford, Damian P Drew

**Affiliations:** 1 Wine Science and Business, School of Agriculture Food and Wine, University of Adelaide, Waite Campus, Urrbrae, South Australia, 5064, Australia; 2 Department of Plant and Environmental Sciences, Faculty of Science, University of Copenhagen, Frederiksberg, 1871, Denmark

**Keywords:** Grapevine, Illumina, Shiraz, RNA-seq, Transcriptome

## Abstract

**Background:**

*Vitis vinifera* berry development is characterised by an initial phase where the fruit is small, hard and acidic, followed by a lag phase known as veraison. In the final phase, berries become larger, softer and sweeter and accumulate an array of organoleptic compounds. Since the physiological and biochemical makeup of grape berries at harvest has a profound impact on the characteristics of wine, there is great interest in characterising the molecular and biophysical changes that occur from flowering through veraison and ripening, including the coordination and temporal regulation of metabolic gene pathways. Advances in deep-sequencing technologies, combined with the availability of increasingly accurate *V. vinifera* genomic and transcriptomic data, have enabled us to carry out RNA-transcript expression analysis on a global scale at key points during berry development.

**Results:**

A total of 162 million 100-base pair reads were generated from pooled *Vitis vinifera* (cv. Shiraz) berries sampled at 3-weeks post-anthesis, 10- and 11-weeks post-anthesis (corresponding to early and late veraison) and at 17-weeks post-anthesis (harvest). Mapping reads from each developmental stage (36-45 million) onto the NCBI RefSeq transcriptome of 23,720 *V. vinifera* mRNAs revealed that at least 75% of these transcripts were detected in each sample. RNA-Seq analysis uncovered 4,185 transcripts that were significantly upregulated at a single developmental stage, including 161 transcription factors. Clustering transcripts according to distinct patterns of transcription revealed coordination in metabolic pathways such as organic acid, stilbene and terpenoid metabolism. From the phenylpropanoid/stilbene biosynthetic pathway at least 46 transcripts were upregulated in ripe berries when compared to veraison and immature berries, and 12 terpene synthases were predominantly detected only in a single sample. Quantitative real-time PCR was used to validate the expression pattern of 12 differentially expressed genes from primary and secondary metabolic pathways.

**Conclusions:**

In this study we report the global transcriptional profile of Shiraz grapes at key stages of development. We have undertaken a comprehensive analysis of gene families contributing to commercially important berry characteristics and present examples of co-regulation and differential gene expression. The data reported here will provide an invaluable resource for the on-going molecular investigation of wine grapes.

## Background

Berry development is a complex process displaying a double sigmoidal growth curve with three distinct phases, including two periods of growth separated by a lag phase during which expansion slows and seeds mature [1]. Cells are established in the first two weeks following flowering, and during the initial growth phase a rapid increase in berry size occurs as a result of cell expansion. The biosynthesis of tannins and hydroxycinnamates and several phenolic compound precursors takes place in the first growth phase [2], and organic acids accumulate in the vacuoles, with malic acid reaching a peak concentration before veraison and then decreasing throughout the second half of the growing season [3]. The short period known as veraison marks the boundary between the lag phase and the third phase of development, and is characterised by the initiation of sugar accumulation, a loss of photosynthetic capacity [4], and the rapid pigmentation of berries by anthocyanins in red grape varieties [1]. High levels of glucose and fructose accumulate after veraison while organic acid levels decrease; the resulting acid to sugar ratio present at harvest is one of the most important contributors to wine sensory characteristics [5]. Towards the end of this third phase of berry development, a number of compounds including terpenes, norisoprenoids, esters and thiols are synthesised [6]. The properties of the berry at harvest, including the final mix of primary and secondary metabolites that accumulate during ripening, are an important determinant of the quality, and therefore value, of the wine produced.

Although the biochemical and physical changes that occur during berry development are well characterised [7,8], the biological processes that control them are less well understood. To a large extent, the biophysical changes that occur during the complex process of grape berry development must be influenced by the presence and activity of metabolic gene pathways. In turn, these metabolic pathways must be controlled by the transcriptional regulation of RNA. Understanding these pathways will give us a greater understanding of the fundamental processes that control berry development, and provide insights into the genetic basis of grape quality that could potentially benefit the wine industry. To this end, several studies have sought to investigate the transcriptional changes that occur during berry development using DNA microarrays [8-14]. Microarray analysis has also been used to investigate differences in gene expression between specific grape tissues [15], and in grapes exposed to a variety of biotic and abiotic stresses or imposed changes to growth conditions [16-23]. Additionally, a collection of microarrays has recently been combined with RNA sequencing to form a grapevine gene expression atlas [24]. The major limitation of most previous microarray studies is that they have generally been limited to interrogating only a portion of the total transcriptome. Many genes are not represented on the microarrays commonly used for grape analysis, while genes that exist in large and highly similar families may give ambiguous expression results due to non-specific hybridisation. Furthermore, the ability of probes to measure transcript abundance is constrained by the accuracy of sequences upon which the array was designed, which is particularly important given the high level of allelic variation in the *V. vinifera* species [25], and the genomic differences between commercial varieties.

The more recent microarray studies have benefited from substantial progress in defining the *V. vinifera* genome in the last five years. The genomes of the variety Pinot Noir and a Pinot Noir-derived variety named PN40024 have been sequenced by two consortia, providing an invaluable resource for studying the molecular mechanisms influencing grape development [25,26]. The *V. vinifera* genome, however, is highly complex and there have been difficulties in producing accurate genomic scaffolds due to its highly heterozygous nature [27]. The PN40024 variety was specifically bred to near-homozygosity to facilitate genomic sequencing and assembly, but most cultivated varieties are extremely heterozygous with allelic differences of up to 13% [25]. The difficulties in genomic assembly have been compounded by the relatively high number of transposons [28], and the fact that some gene families are highly repeated and interspersed with numerous pseudo genes [29]. Nevertheless, algorithmic predictions of the grapevine transcriptome, combined with a large amount of expressed sequence tag (EST) data, have been used to design and annotate microarray platforms for the interrogation of grape berry transcripts. Although valuable data on transcriptional regulation in grapes has been reported, the aforementioned technical limitations of microarrays have limited their level of coverage.

Massively parallel RNA deep-sequencing represents an alternative technological platform for investigating transcriptional regulation. It enables the precise elucidation of transcripts present within a particular sample, and can be used to calculate gene expression based on absolute transcript abundance [30]. In the single reported grapevine study to date, Zenoni *et al*. (2010) generated RNA sequencing data from *Vitis vinifera* (cv. Corvina), and provided an initial overview of the complex process of gene regulation during berry development [31]. Due to the rapidly advancing technology of next generation sequencing, the amount of sequencing data that can be generated in a single experiment has increased dramatically in recent years, as has the length of the sequencing reads. This has led to a greater level of transcriptome coverage and an increase in the specificity, and therefore accuracy, when mapping sequencing reads. Importantly, continuous incremental advances in defining the grapevine transcriptome in the form of functional annotation [32,33] and gene ontology assignment [34] now enable an accurate description of the functional roles of the majority of *V. vinifera* genes. In this report, we use the latest RNA sequencing technology to carry out a comprehensive analysis of the global transcriptional profile of grape berries (cv. Shiraz) during the immature green phase, at early and late veraison, and in ripe berries. We investigate the suitability of a number of reference transcriptomes for RNA-Seq analysis in grapevine, validate a number of the transcriptional changes observed using quantitative real-time PCR, and describe the biological processes that are enriched in differentially regulated gene clusters.

## Results and discussion

### Grape sampling and development

**Figure 1:**
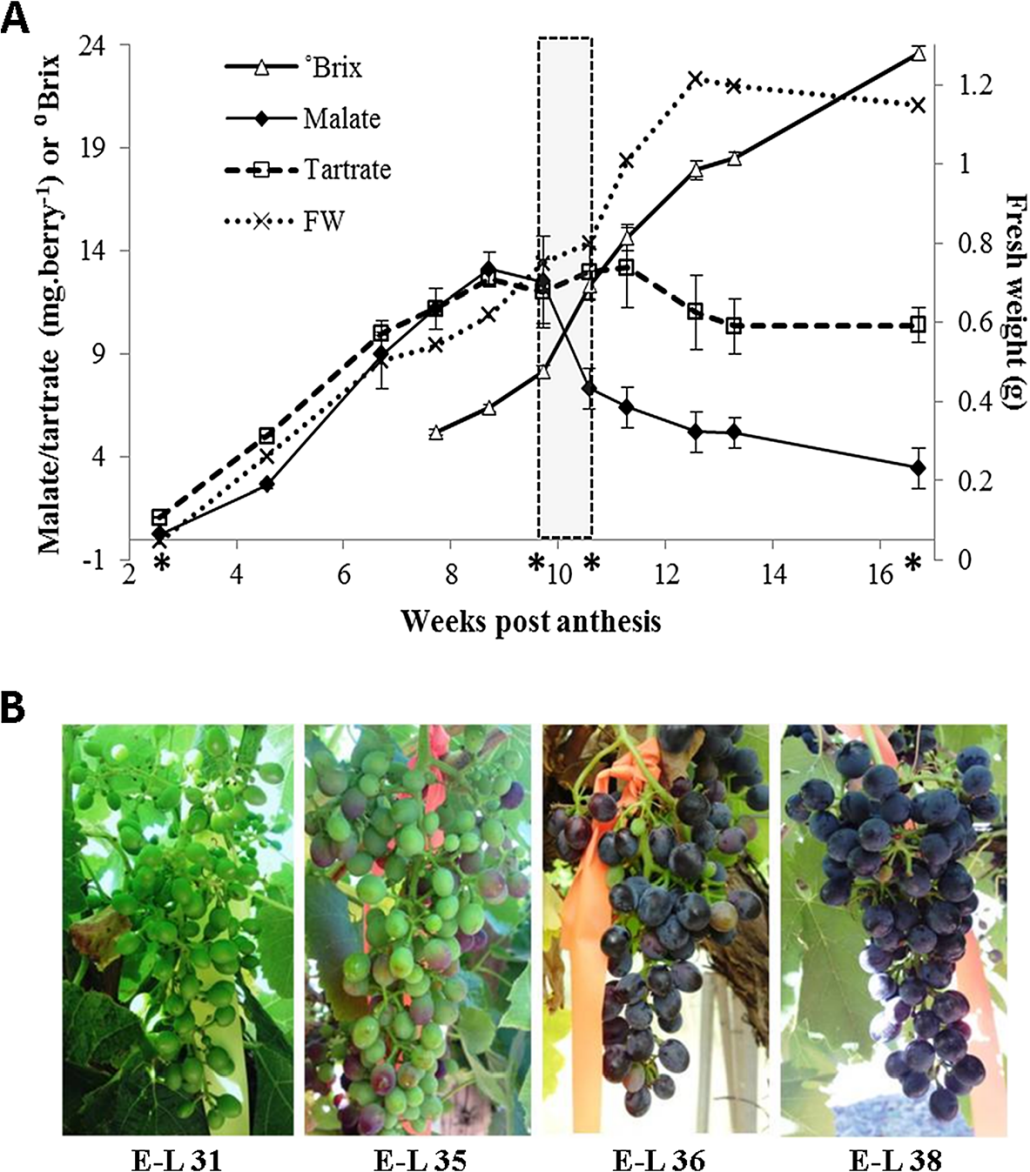
**Shiraz berry developmental measurements. A**. ^0^Brix, tartrate and malate levels are presented as the mean of three biological replicates (± S.E.M.). Veraison is highlighted by a dashed box, and samples from which RNA was submitted for transcriptome sequencing are indicated with an asterisk below the x-axis. **B**. Images of representative bunches at the time-points selected for sequencing, corresponding to developmental stages E-L 31, 35, 36 and 38 and referred to in the text as young berries, early-veraison, late-veraison and ripe berries.

Berries from *V. vinifera* (cv. Shiraz) were sampled at 7 to 14-day intervals throughout the growing season, with the shorter intervals occurring in the period coinciding with the expected time of veraison. Fruit development was monitored by the measurement of fresh weight and malic acid and tartaric acid content per berry in samples from 3 weeks post-anthesis until harvest at 17 weeks, and total soluble solids (degrees Brix; °Bx) measurements were taken from 7 weeks until harvest. The fresh weight of berries increased throughout the season, with a slowing of growth observed at about 9 weeks followed by a rapid increase in fresh weight from 10 to 13 weeks. Malic acid content in berries increased early in the season and peaked at approximately 9 weeks post-anthesis (Figure 1A). From 9 to 12 weeks the malic acid content per berry dropped rapidly and continued to decrease until harvest. Total soluble solids, as measured by °Bx, increased consistently between each sampling point, with the most rapid increase occurring between weeks 10 and 11 (Figure 1A). The end of the herbaceous plateau, decreasing malic acid content and rapidly increasing °Bx are examples of the physiological changes that characterise veraison, which is most easily recognised in red grape varieties by the development of pigment over a relatively short period of time (Figure 1B). Given our interest in the transcriptional changes that may be involved in regulating grape development, based on these data we chose to carry out global mRNA sequencing on samples from 3-, 10-, 11- and 17-weeks post-anthesis, corresponding to stage E-L 31, 35, 36 and 38 on the modified E-L system [35].

### Illumina HiSeq mRNA sequencing

Prior to sequencing, RNA integrity numbers were determined for poly(A) mRNA isolated from each of the four developmental stages using a Bioanalyzer 2100 (Agilent Technologies). Calculated values of 9.20, 9.30, 8.60 and 9.30, respectively, indicated that little degradation of mRNA had occurred during extraction or subsequent processing, suggesting full-length or near full-length mRNAs were likely to be present and predominant. Each of the four mRNA samples was indexed with unique nucleic acid identifiers and sequenced on a single lane of an Illumina HiSeq 2000 instrument. In total, 162,353,167 reads of 100 bp were generated, giving a total of over 16 billion nucleotides of sequence data. This compares favourably with the 2.2 billion nucleotides of sequence data consisting of 59 million 36-44 bp reads obtained for the previous reported grape berry transcriptome sequencing project [31]. In addition to the almost 8-fold higher sequence coverage, the 3-fold longer read length enabled a much greater degree of accuracy when mapping to reference genomes or transcriptomes. De-multiplexing using the unique identifiers revealed that our data consisted of 35,656,501 reads from young berries, 39,624,765 reads from pre-veraison berries, 42,052,446 reads from post-veraison berries and 45,019,455 reads from ripe berries. This provided almost 10-fold higher sequencing read number than a recent Illumina-based transcriptome analysis of fruit development in Chinese bayberry, which investigated gene regulation based on 5.3 million 90 bp reads [36].

### Investigation of mapping references for RNA-Seq analysis

We first investigated a number of reference transcript collections in order to determine whether a comprehensive and accurate description of berry transcriptional profiles could be developed by mapping and counting the reads generated through Illumina sequencing against predicted mRNA transcripts. Two independent groups have generated near-complete *V. vinifera* (cv. Pinot Noir and cv. PN40024) consensus genome assemblies [25,26], and the former of these groups, the French-Italian Public Consortium for Grapevine Genome Characterization, has released two publically accessible versions of the complete *V. vinifera* genome at 8x and 12x coverage (available from http://www.genoscope.cns.fr/externe/Download/Projets/Projet_ML/data/) [25]. Algorithmic predictions of mRNA transcriptomes based on this data and using the GAZE computational framework resulted in the prediction of 30,434 or 26,346 transcripts from the 8x and 12x genome assemblies, respectively, and provided the first two datasets to which we mapped and counted our sequencing reads. In addition, the National Center for Biotechnology Information reference sequence (NCBI RefSeq) database provided an alternative resource of predicted *V. vinifera* mRNA transcripts [33]. While NCBI RefSeq transcripts are based on the 12x genome of Jaillon *et al.* (2007), they are predicted by the Gnomon algorithm, which draws on supporting evidence such as ESTs and alignments to orthologous transcripts and proteins, and are manually curated and continually updated [37]. The NCBI RefSeq nucleotide collection, consisting of 23,720 annotated transcripts, comprised the third reference dataset for our mapping reference.

The use of an mRNA transcript collection as a mapping reference is an alternative approach to that taken by Zenoni *et al.* (2010), who instead used the draft consensus genome reported by Jaillon *et al.* (2007) as a reference. Mapping of mRNA sequencing reads against genomic scaffolds requires prior knowledge of gene structure, or can be carried out through the use of algorithmic predictions of splice junctions [38]. However, given the complexity of the draft consensus genome, its high reported heterozygosity, and the difference in grape variety under investigation, we chose instead to focus on the transcribed component for our analysis. When carrying out RNA-Seq mapping, we excluded reads with greater than two ambiguous nucleotides, as well as the small proportion of reads that were less than 60 bp in length. This resulted in a total pool of 148,945,405 reads from the four developmental stages that were counted for transcript mapping (Table 1). We used a similarity threshold of 98%, and set the minimum proportion of the read that must match a reference at 0.5 to allow for the mapping of reads that included up to 50 bp of UTR in cases where this was not included as part of the mapping reference. Somewhat surprisingly only about 58.0% of our sequencing reads could be mapped against the Genoscope 8x or 12x predicted transcriptomes (Table 1). In contrast, 83.6% could be mapped to the NCBI RefSeq collection. The proportion of our sequencing reads mapping to the NCBI RefSeq collection is actually higher than the proportion of shorter reads that were previously mapped to the *Vitis vinifera* draft consensus genomic scaffolds [31], highlighting the suitability of our chosen mapping reference.

**Table 1:** Comparison of transcriptome datasets as a reference for RNA-Seq analysis

**Developmental stage**	**Counted reads**	**Genoscope 8x (30434 transcripts)**	**Genoscope 12x (26346 transcripts)**	**NCBI RefSeq (23720 transcripts)**
**Young berries**	32 283 153	17 127 563 (53.0)	16 340 511 (50.6)	24 972 894 (77.3)
**Early-veraison**	36 280 465	21 111 334 (58.2)	22 423 774 (56.6)	30 695 559 (84.6)
**Late-veraison**	38 434 765	23 572 898 (61.3)	22 522 747 (58.6)	33 605 499 (87.4)
**Ripe berries**	41 947 022	24 606 233 (59.6)	25 516 490 (58.1)	35 251 865 (85.4)
**Total**	148 945 405	86 418 028 (58.0)	86 803 522 (58.3)	124 525 817 (83.6)

Number of Illumina HiSeq sequencing reads from each developmental stage mapped to selected reference mRNA transcript collections. The percentage of counted reads that were mapped is presented in parenthesis.

Although the use of a nucleotide mapping reference means the genes investigated in our analysis are determined by pre-existing transcriptomic data, the high percentage of reads mapped under high-stringency conditions indicated a high level of coverage of actual transcribed sequences. Additionally, the use of the NCBI RefSeq nucleotide collection facilitates direct comparison of mapped transcripts with well-described gene functions and manually updated annotations. The method employed by Bellin and co-workers [23], whereby pyrosequencing of 3’ cDNA ends and *de novo* contig assembly was used to create a library of unigenes for microarray design, represents an approach to transcriptome analysis that overcomes the issue of predetermined transcript data. This combination of next generation sequencing and microarray generation will be particularly valuable for non-model species for which genomic information is limited. However, in the case of *V. vinifera*, for which relatively well-annotated genomic and transcriptomic data are available, the use of a nucleotide mapping reference represents a convenient technique that allows the utilisation of annotations detailed and updated on NCBI.

While the Genoscope 8x predicted transcriptome contained 30,434 sequences, the NCBI RefSeq dataset consisted of only 23,720 sequences. Given that a much higher proportion of our Illumina sequencing reads mapped to the RefSeq dataset than to the Genoscope dataset (83.6% compared to 58%; Table 1), it was considered unlikely that the difference of almost 7,000 transcripts was simply a result of absent genes from the RefSeq mRNA collection. A batch BLAST search using each of the Genoscope predicted transcripts as a query against the RefSeq mRNA dataset revealed that about 28,000 (92%) of the 30,434 Genoscope transcripts had a hit in the RefSeq dataset with an e-value approaching zero (data not shown). However, this included numerous duplicates where multiple short Genoscope transcripts were matched to a single RefSeq transcript. When these duplicates were removed, a list of approximately 20,000 accessions remained. Furthermore, investigation of a subset of Genoscope transcripts that had no BLAST hit within the RefSeq transcript collection revealed that many of these predicted transcripts were 100 nucleotides or less, and were probably partial gene sequences that did not have a significant match because of their length. We therefore propose that the widely used *V. vinifera* transcriptome prediction from Genoscope contains multiple redundant accessions that have probably come about as a result of incorrect assignment of splice junctions. This analysis, combined with a higher proportion of mapped sequence reads, indicated that the manually curated NCBI RefSeq dataset is the most comprehensive and accurate collection of *V. vinifera* mRNAs currently available, and we proceeded to use this set of reference transcripts to investigate mRNA abundance and transcriptional regulation in grape berries.

### Transcript expression analysis

Transcript abundance was determined by the calculation of Reads Per Kilobase of exon per Million mapped reads (RPKM) [30]. Unique reads were counted to matching transcripts, and non-specifically mapped reads were allocated on a proportional basis relative to the number of unique reads already mapped. A limitation of this method is in the case of differentiating between recently duplicated isogenes with coding sequence exceeding 98% identity, and thus expression values in these instances should not be considered definitive. A method of measuring differences in expression between highly similar isogenes by microarray analysis of non-coding regions has been described for a subset of the *V. vinifera* genome [9]. The accuracy of the RPKM method for calculating transcript expression is also impacted in cases where full-length sequences are not transcribed due to premature stop codons, structural variation or differences between the mapping reference and the actual transcript. Nevertheless, with these limitations in mind, out of the 23,720 predicted transcripts in the NCBI RefSeq mRNA collection, 17,942-18,729 transcripts could be detected at each developmental stage (Table 2). For these data the lower limit for detection was designated to be an RPKM of 0.5, or if the RPKM value was less than 0.5 then a minimum of five uniquely matched reads (at greater than 98% identity over 100 bp) were required for a transcript to be considered present. Only 3,208 out of 23,720 transcripts, approximately 13.5%, did not meet these criteria for detection in any of the four developmental stages (Additional file 1: Table S2). To put the RPKM values from our study in perspective, a value of 0.5 corresponds to an average transcript coverage of 2, or about 2000 bp of sequencing read coverage for a 1000 bp transcript. In a recent comparable study, Zenoni et al. (2010) estimated that their statistical analysis would be reliable when applied to genes with 6 mapped 36-44 bp reads covering 200 bp of transcript, which could otherwise be expressed as average coverage of approximately one [31]. A benefit of mapping 100 bp reads compared with the shorter reads generated in the work of Zenoni et al. (2010) is the increased specificity, and thus accuracy, of transcript expression analysis. Allowing for two mismatches, a 36 bp read maps with only 94% identity, inevitably leading to alignment with multiple locations, especially in the case of closely related multi-gene families. In the current study, approximately 85% of reads that were matched at 98% identity or greater were aligned to a single location in our reference dataset (data not shown), compared with 66.6% matched to unique locations by Zenoni et al. (2010) [31].

**Table 2:** Transcript abundance measurements at each developmental stage

	**Young berries**	**Early-veraison**	**Late-veraison**	**Ripe**
**RPKM > 200**	679	477	466	411
**RPKM 10-200**	7 458	8 179	7 582	7 908
**RPKM 0.5-10**	8 994	7 994	8 016	8 327
**RPKM 0-0.5 (unique reads > 5)**	1 499	1 717	1 878	2 083
**Total detected**	18 720	18 367	17 942	18 729

Numbers of transcripts from the NCBI *Vitis vinifera* RefSeq dataset detected at various levels of abundance at each time-point, as calculated by reads per kilobase of exon per million reads (RPKM).

The fact that 70-80% of the NCBI RefSeq mRNA transcripts for *V. vinifera* could be detected in each of our samples, and 86.5% of transcripts could be detected in at least one sample, is a testament to the power of RNA-Seq analysis as a technique for transcriptional studies, compared with microarray analyses in which probes have historically covered a limited portion of the *V. vinifera* transcriptome. Furthermore, the depth of our Illumina sequencing data enabled us to investigate the expression of transcripts that are present at extremely variable absolute levels. For example, the lower limit for detection for which we report transcript regulation in this study, corresponding to an RPKM of 0.5 (Table 2), represents transcripts with an absolute abundance 90,000-fold lower than the most abundant transcript in ripe berries, XM_002284998.2, which had an RPKM of 44,999. The mRNA XM_002284998.2 (corresponding to Genoscope accession GSVIVT00020222001), which encodes an uncharacterised proline-rich protein of 236 amino acids, with sequence similarity to extensin related cell-wall proteins, accounted for an impressive 4.5% of the sequencing reads generated from ripe berries. This highlights one of the benefits of RNA-Seq expression analysis over microarray analysis in uncovering transcripts that may be of interest. Microarrays determine changes in the relative expression of transcripts between two or more samples, but do not provide accurate quantitative data on the absolute level of expression of a transcript within any given RNA sample due to differences in probe binding specificity and efficiency. As a resource for grapevine researchers, we present the absolute expression levels of all transcripts in each of the four developmental stages investigated here in Additional file 1: Table S1, alongside the closest matching Genoscope accession and the functional annotation of the encoded protein.

### Global comparison with microarray analysis of developing grape

**Figure 2:**
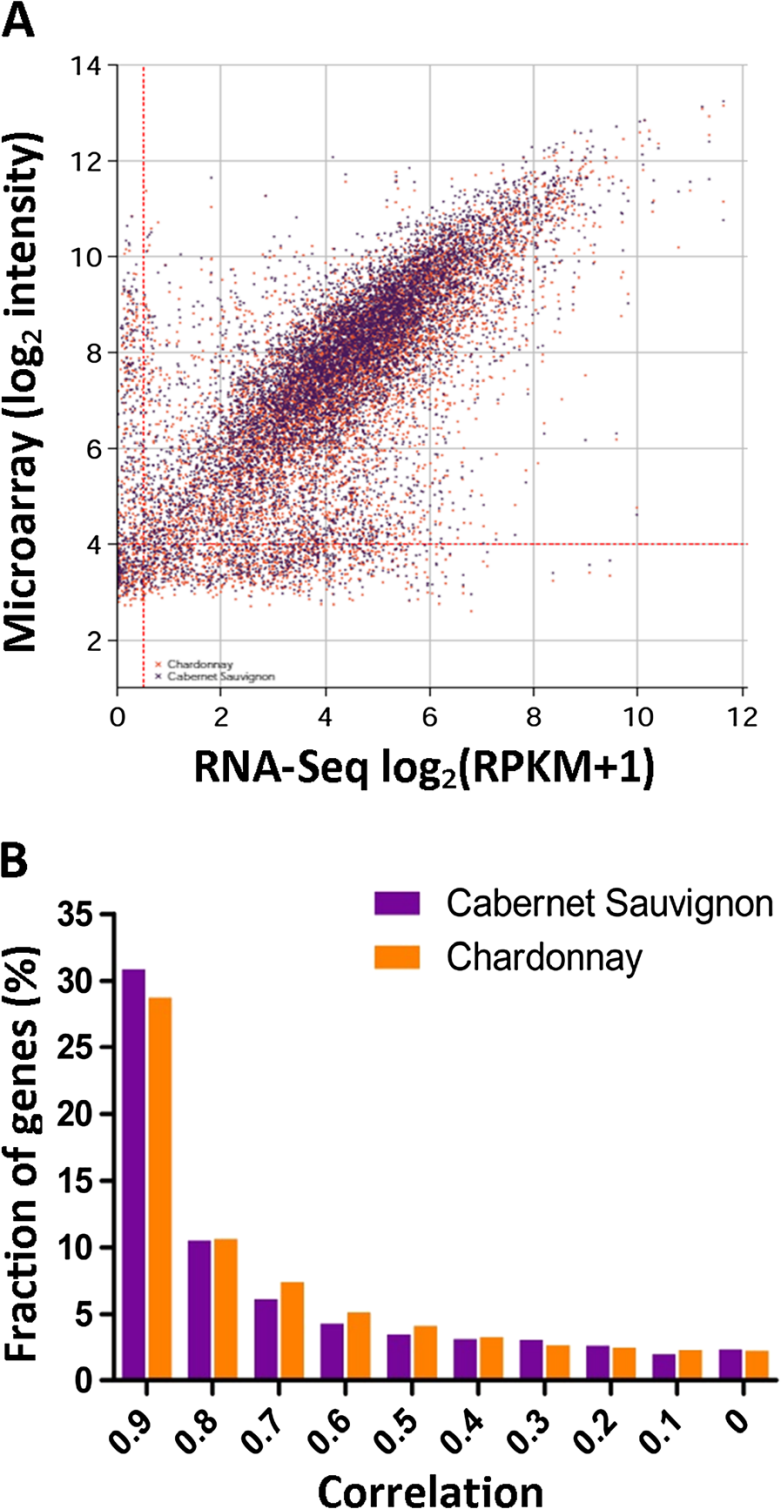
**Global comparison of RNA-Seq and microarray analysis of transcript expression in developing grape. A**. Comparison of microarray probeset intensities for developing Cabernet Sauvignon (purple) and Chardonnay (orange) [8] with transcript abundance for the corresponding genes measured in our study as expressed by log2 (RPKM + 1). Expression values charted here consists of the mean of four developmental stages corresponding to E-L 31, E-L 35, E-L 36 and E-L 38, and give Spearman correlation coefficients of ρ = 0.73 and ρ = 0.72 for Cabernet Sauvignon and Chardonnay, respectively. Dashed lines represent the cut-off whereby genes are not considered expressed in either platform and are not included in the calculation of correlation coefficients. **B**. Histogram showing the distribution of correlated genes during berry development. Mapped transcripts having a Spearman correlation between correlation thresholds were counted from a total of 7189 unique transcripts measured by both platforms.

Given the surfeit of literature reporting transcript expression in grapes based on the microarray platform, we investigated the correlation between our measurements of mRNA transcript abundance based on RNA-Seq analysis and gene expression levels previously reported at equivalent developmental stages based on the Affymetrix GeneChip. Deluc *et al*. (2007) investigated transcriptional regulation in developing grapes of *Vitis vinifera* (cv. Cabernet Sauvignon and cv. Chardonnay) at a number of developmental stages, including those corresponding to E-L 31, E-L 35, E-L 36 and E-L 38 [8]. Although the varieties of grapes investigated by Deluc *et al.* (2007) differed from the variety studied in this report, we predicted that a majority of transcripts should exhibit similar relative abundances within each stage of berry development investigated here. For this comparison, we considered only GeneChip probesets for which the originating EST has an exact BLASTn match (e-value = 0) in the NCBI RefSeq dataset, and discarded probesets that cross-hybridised with multiple transcripts. Transcripts that were expressed at low or background level in either microarray or RNA-Seq analysis were also removed, leaving 6899 and 6848 transcripts for Cabernet Sauvignon and Chardonnay, respectively. For this subset of transcripts, the correlation between our RNA-Seq analysis and their expression in the corresponding developmental stages reported by Deluc *et al.* (2007) was approximately ρ = 0.73 for Cabernet Sauvignon and ρ = 0.72 for Chardonnay (Figure 2A). These relatively high correlation coefficients indicate that the absolute transcript expression levels we report within a single developmental stage of berry based on RNA-Seq give similar results to previous data generated by the Affymetrix GeneChip microarray.

We also examined the correlation between the pattern of relative expression formed by the four developmental stages examined in our RNA-Seq analysis compared with the equivalent expression pattern as measured by microarray [8]. We found that the expression patterns of a majority of transcripts in both Cabernet Sauvignon and Chardonnay were positively correlated with our Shiraz RNA-Seq analysis. This included about 30% of transcripts for which the patterns of expression measured by the two platforms were extremely highly correlated with a ρ ≥ 0.9 (Figure 2B). The majority of transcript expression patterns were positively correlated to some degree, with about 57% of transcripts exhibiting a medium to high correlation of ρ ≥ 0.6. The high correlation between differential expression of transcripts reported here and the expression patterns previously measured by microarray, goes some way towards validating the utility of our data for investigating transcriptional regulation during grape development.

### Highly expressed transcripts throughout grape development

Approximately 400-700 transcripts from each developmental stage had an RPKM value of 200 or greater (Table 2), and as such represented the top 1.7-2.9% of mRNAs by absolute expression level. Of these transcripts, 153 had an RPKM of over 200 in all four samples under investigation (Additional file 1: Table S2). In addition to 31 uncharacterised proteins, the products of these highly expressed transcripts included a number of proteins that would generally be expected to be highly expressed in most cell types. These included 16 ribosomal proteins, 12 translation initiation and elongation factors, 8 proteins involved in amino acid metabolism, 6 glycolysis pathway enzymes, 2 catalase isoforms, 2 actin-related proteins, 2 vacuolar proton ATPases, super-oxide dismutase and RuBisCo. It is interesting to note that 147 out of 153 of these highly expressed transcripts have a matching Affymetrix probeset ID, despite the fact that only 34% of the RefSeq sequences are represented on the microarray. This is likely due to the fact that the design of microarray probesets was based predominantly on EST data, in which highly expressed transcripts are inherently over-represented. Conversely, of the 3,208 transcripts that were not detected in any of our four samples, only 239 (7.5%) are represented by an Affymetrix probe (Additional file 1: Table S3).

Given the apparent constitutively high level of transcription for the genes mentioned above, it could be suggested that they are likely to play an important role in biological processes occurring during berry development. However, since we have not investigated other tissue from grapevine in this study, we do not present evidence that the transcripts are *specifically* involved in berry development relative to other grapevine tissues. Nevertheless, some potential berry-related genes can be seen within this list. Since high levels of malic acid are synthesized in berries until veraison, and both the concentration and absolute level rapidly decrease after veraison [3], it is not surprising that a cytoplasmic malate dehydrogenase (MDH; XM_002278672.2), which catalyses the reversible conversion of oxaloacetate to malate, was highly abundant at all stages. Another functionally annotated cytoplasmic MDH enzyme (XM_0022786002.2) was similarly abundant, while the third isoform (XM_002277507.2) was approximately 50-fold less abundant. Immediately adjacent to MDH in the citric acid cycle, citrate synthase (XM_002278145.2) was also one of the consistently abundant transcripts in our data. Given that malate accumulates in the berry vacuole where it is compartmentally separated from the citric acid cycle enzymes, the highly abundant putative malate carrier protein (XM_002285686.1) may warrant further functional investigation.

Another abundant, putatively berry-specific gene was a chalcone synthase isoform (CHS2; XM_002263983.1), which is a potential upstream regulator of a number of phenolic secondary metabolites, including tannins, anthocyanins and flavonols. In contrast, CHS1 has previously been shown to be developmentally regulated with highest expression occurring in young berries [39], a result that is consistent with our RNA-Seq analysis (Additional file 1: Table S1). The finding that two genes putatively involved in the metabolism of alpha-linolenic acid (XM_002272955.2 and XM_002285538.2) were highly abundant is interesting since n-3 fatty acids such as linolenic have only been found to be present in grapes at extremely low concentrations [40]. Thus, the high expression level of these two transcripts in grapes suggests further characterisation may be required to determine their true functional activity. One isoform of hydroxymethylgutaryl-CoA synthase (XM_002282398.2) was highly abundant, while the other (XM_002262655.2) was not detected in any of our samples, highlighting the importance of isoform-specific expression data.

### Specifically up-regulated transcripts

We used our quantitative expression analysis to investigate genes that are transcriptionally regulated during specific stages of berry development. First, we investigated genes that were more highly expressed in a single developmental stage when compared with their expression levels in each of the other three stages. To account for low and zero values in our data while still identifying biologically significant changes, differences were calculated relative to an RPKM of 0.1 when calculating fold changes from RPKM values of less than 0.1. Thus, the difference between 0.02 and 1.00 was considered a 10-fold or greater increase, but not a 50-fold increase. This went some way towards discarding unrealistically high expression changes that are an unavoidable consequence of data that incorporates values approaching and including zero. In total, there were 4,185 transcripts that exhibited 3-fold or greater increased expression at a single developmental stage compared with all other stages (Table 3). A relatively low number of these transcripts were specifically up-regulated at early- or late-veraison (194 and 59 transcripts, respectively). The low number of genes specifically regulated at these time points is probably due to the fact that only a single week separated the two samples. Furthermore, in order to capture a representative biological selection of transcripts at each time-point, RNA for Illumina sequencing was purified from tissue consisting of 20 berries collected from 10 bunches that had been monitored from the beginning of the growing season and tagged at 50% cap-fall (see Methods). Since it takes approximately one week for a single bunch to develop from 0% to 100% cap-fall, it could be argued that individual grapes on any given bunch are separated by up to a week in their absolute developmental age. This biological variation within each of our early- and late- veraison stages could have masked transcriptional regulation events that take place over the relatively short one-week period during which pigmentation occurs (Figure 2, E-L 35 to E-L 36). Also, it has been reported that major changes in gene expression can occur over as little as 24 hours, and that this happens before changes in pH, sugars and berry colouring can be observed [9]. Therefore, an in-depth analysis of genes that are differentially expressed between young berries and veraison, and between veraison and full-ripening, could yield more useful information about global changes in metabolism. With this in mind, we also generated data on the number of transcripts that are specifically up-regulated at *both* the time-points taken around veraison (E-L 35 and E-L 36), compared with their expression level in young or ripe berries (Table 3 – ‘veraison’). A complete list of all 4,185 transcripts that are specifically up-regulated 3-fold or more at a single developmental stage corresponding to the transcripts counted in Table 3, and an additional 122 transcripts that are specifically over-expressed during both early- and late-veraison, is provided in Additional file 2.

**Table 3:** Transcripts over-expressed at a single developmental stage

	**Young berries (E-L 31)**	**Early-veraison (E-L 35)**	**Late-veraison (E-L 36)**	**Harvest (E-L 38)**	**Total**	**Veraison**
**> 50-fold higher**	282	1	0	65	348	3
**10-50 fold higher**	789	31	1	175	996	60
**3-10 fold higher**	1 814	162	58	807	2 841	312
**Total**	2 885	194	59	1 047	4 185	375

The numbers of transcripts significantly up-regulated in berries at a single developmental stage relative to all other samples. Fold changes are calculated compared with a minimum RPKM value of 0.1. Given the similarity in transcript expression patterns between early and late-veraison (E-L 35 and E-L 36), the relative expression of transcripts in both of these stages compared with E-L 31 and E-L 38 are reported in the separated column “veraison”.

Given the observed similarity between RPKM data from early- and late-veraison samples, we investigated the overall correlation between these two stages in order to estimate the technical variation within our experiment. The Pearson’s correlation coefficient for global transcript expression between these two stages (E-L 35 and E-L 36) was ρ = 0.99, which is equivalent to the correlation expected for high quality technical replicates of the same RNA sample [41]. Additionally, only about 2% of genes exhibited a log_2_ transcript abundance difference of greater than 1.6 (equating to about 3-fold) between these stages (data not shown), which could be explained as a conservative description of the transcripts truly differentially expressed between early- and late-veraison. Thus, although it will be desirable to analyse global transcript abundance from more highly separated time-points around veraison when investigating developmental regulation in future studies, we were able to use these two samples as de facto replicates in order to demonstrate that our RPKM expression data was reproducible and that 3-fold and greater changes in abundance were very unlikely to be the result of technical variation.

One of the clearest findings from an analysis of transcripts that were highly over-expressed at a single stage was the large number of biological processes activated in young berries (stage E-L 31) that do not occur during veraison or in ripe berries. In young berries, 2,885 of the 23,720 investigated transcripts were specifically overexpressed relative to all other time-points, while 1,047 were specifically up-regulated in ripe berries (Table 3). The 2,885 transcripts that were at least 3-fold up-regulated in immature berries represented a significant 12% of the total grapevine predicted transcriptome, or approximately 15% of the transcripts expressed in berries. A portion of these genes were extremely highly up-regulated with 282 transcripts up-regulated over 50-fold in young berries compared with expression levels in any other sample. Many of the transcripts more highly expressed in young berries can be linked with the photosynthetic capacity of grapes during early stages of development, which decreases dramatically during ripening [4]. For example, 18 of the 20 annotated chlorophyll a-b binding proteins from grapevine were amongst these 2,885 transcripts, as were 12 out of 15 photosystem I reaction center subunit-encoding transcripts, two of the three transcripts encoding the photosystem II reaction center W and transcripts for photosystem II 5kDa and 22kDa core-complex proteins (data is searchable in Additional file 2). Transcripts encoding enzymes from other metabolic pathways reported to occur early in grape berry development were also highly over represented in the list, such as genes involved in the biosynthesis of tannin precursors. These include anthocyanidin reductase, leucanthocyanidin reductase, and five anthocyanidin 3-O-glucosyltransferases, which stabilise anthocyanins through glycosylation [42].

Transcription factors are of particular interest given their ability to control the expression of numerous genes, and thus their ability to regulate biological pathways and developmental processes. There were 26 annotated transcription factors specifically over-expressed 50-fold or greater in young berries, most of which had zero or negligible expression at the other stages investigated here (Table 4). These included nine transcripts encoding ethylene-responsive transcription factor (ERF) 5-like proteins. We found that a further four ERF5-like transcripts were specifically up-regulated between 10- and 50-fold in young berries (Additional file 2). Combined, these transcripts comprised 13 of the 17 annotated ERF5-like genes, while the remaining four ERF5-like transcripts were all up-regulated 2- to 3-fold in young berries. Six other transcription factors that were 50-fold or greater up-regulated in young berries are annotated as ethylene-responsive transcription factors, including two ERF17s, ERF7, ERF23, ERF109 and ERF-WIN1 (Table 4). Whether these families of transcription factors are responsive to ethylene in grapes has not been established, and it is important to remember that the majority of functional annotations are made based on sequence similarity to proteins from other species, predominantly Arabidopsis. Indeed, while ethylene signalling is known to play an important role in the ripening of climacteric fruit, the precise role of ethylene signalling, if any, in grape development remains an active area of research [43,44]. Nevertheless, the high degree of transcriptional specificity of these families of transcription factors is a strong indication that they are responsible for regulating biological processes that occur early during grape berry development.

**Table 4:** Specifically up-regulated transcription factors

**RefSeq Accession**	**Closest Genoscope match**	**Affymetrix Probeset ID**	**Young berries**	**Early-veraison**	**Late-veraison**	**Ripe berries**	**Encoded protein annotation**
XM_002282012.2	GSVIVT00014253001		**125.18**	0.51	0.76	0.35	Ethylene-responsive Transcription factor 5
XM_002281930.1	GSVIVT00014247001		**121.59**	1.73	1.03	0.29	Ethylene-responsive Transcription factor 5
XM_002282133.2	GSVIVT00036589001		**114.34**	0.52	0.60	0.45	Ethylene-responsive Transcription factor ERF109
XM_002276536.2	GSVIVT00016398001	1608812_at	**105.10**	0.15	0.27	0.13	Ethylene-responsive Transcription factor ERF017-like
XM_002282279.1	GSVIVT00023866001		**90.51**	0.24	0.22	0.86	Ethylene-responsive Transcription factor 7-like
XM_002281911.2	GSVIVT00014244001		**83.05**	0.51	0.47	0.14	Ethylene-responsive Transcription factor 5-like
XM_002281777.2	GSVIVT00014237001	1613698_at	**80.37**	1.36	0.50	0.28	Ethylene-responsive Transcription factor 5
XM_002281895.2	GSVIVT00014242001	1619600_at	**73.43**	0.80	0.53	0.19	Ethylene-responsive Transcription factor 5
XM_002268377.2	GSVIVT00000349001		**46.99**	0.24	0.15	0.06	Ethylene-responsive Transcription factor WIN1-like
XM_002281047.2	GSVIVT00022870001	1616185_at	**39.00**	0.11	0.03	0.17	Transcription factor bHLH96-like
XM_002282131.1	GSVIVT00014256001		**38.48**	0.44	0.23	0.13	Ethylene-responsive Transcription factor 5
XM_002280334.1	GSVIVT00032308001		**36.98**	0.00	0.00	0.05	Ethylene-responsive Transcription factor ERF017
XM_002281876.2	GSVIVT00014240001		**31.92**	0.20	0.40	0.10	Ethylene-responsive Transcription factor 5
XM_002281835.2	GSVIVT00014238001	1613799_at	**29.46**	0.30	0.09	0.00	Ethylene-responsive Transcription factor 5
XM_002284201.1	GSVIVT00014754001		**29.28**	0.35	0.21	0.20	Transcriptional activator Myb
XM_002263558.1	GSVIVT00006679001		**22.72**	0.12	0.00	0.03	Transcription factor RAX1
XM_002263958.2	GSVIVT00008628001		**19.81**	0.04	0.00	0.04	Ethylene-responsive Transcription factor ERF023
XM_002268533.2	GSVIVT00000129001		**18.68**	0.08	0.07	0.16	Transcription factor TCP15-like
XM_002283709.1	GSVIVT00032414001	1609286_at	**18.29**	0.13	0.05	0.11	GATA Transcription factor 9
XM_002274170.1	GSVIVT00034800001		**11.10**	0.04	0.00	0.03	Transcriptional activator Myb
XM_002276513.1	GSVIVT00037009001		**9.52**	0.08	0.03	0.13	Transcription factor bHLH135
XM_003633976.1	GSVIVT00014248001		**8.02**	0.00	0.09	0.03	Ethylene-responsive Transcription factor 5-like
XM_002283058.1	GSVIVT00020927001	1613614_at	**7.56**	0.11	0.00	0.00	Transcription factor bHLH135
XM_002276926.1	GSVIVT00029219001		**7.43**	0.00	0.00	0.00	Transcription repressor MYB4
XM_002274226.2	GSVIVT00018597001		**5.91**	0.07	0.00	0.06	Transcription factor bHLH118-like
XM_002284800.1	GSVIVT00014836001		**5.31**	0.07	0.04	0.04	Heat stress Transcription factor B-4
XM_003632349.1	GSVIVT00001240001	1621346_at;	0.10	**50.36**	**25.69**	7.32	B3 domain-containing transcription factor ABI3-like
XM_002275111.1	GSVIVT00025350001		0.14	**30.00**	**15.82**	4.91	Transcription factor HBP-1b(c1)-like
XM_003632364.1			0.00	**4.66**	**4.85**	0.89	Transcription factor UPBEAT1-like
XM_002283723.2			0.00	**2.52**	**0.93**	0.06	myb family transcription factor APL-like
XM_002272753.2	GSVIVT00031144001	1609798_at	30.57	50.29	34.79	**155.93**	Trihelix transcription factor GTL2-like
XM_002276158.2	GSVIVT00017225001	1610832_at	8.91	19.82	17.88	**67.73**	Probable WRKY transcription factor 32
XM_002281158.1	GSVIVT00028232001		5.78	13.73	18.95	**61.77**	Probable WRKY transcription factor 47-like
XM_003635597.1		1611921_at	7.36	16.93	13.95	**59.56**	GATA transcription factor 26-like
XM_002269660.2	GSVIVT00025898001		1.14	9.70	11.70	**57.63**	WRKY transcription factor 6-like
XM_002273307.2	GSVIVT00013494001	1620116_at	9.62	13.68	10.78	**44.93**	GATA transcription factor 26
XM_002275540.1	GSVIVT00002773001	1607465_at	1.34	2.26	3.46	**43.23**	Probable WRKY transcription factor 57
XM_002274248.2	GSVIVT00033300001		0.22	0.67	1.28	**23.91**	Ethylene-responsive transcription factor ERF003
XM_003631122.1	GSVIVT00030611001	1608728_at	6.36	6.37	4.36	**19.42**	Heat stress transcription factor A-8-like
XM_002267778.1	GSVIVT00006201001	1609629_at	0.76	0.23	0.36	**10.56**	Ethylene-responsive transcription factor ERF113
XM_002283591.1	GSVIVT00024804001		0.10	0.88	1.21	**9.53**	Ethylene-responsive transcription factor RAP2-11
XM_002275357.2	GSVIVT00003416001	1622116_at	0.59	0.46	0.10	**4.36**	Transcription factor bHLH144 isoform 2
XM_003632808.1	GSVIVT00034227001		0.14	0.88	0.45	**4.20**	Transcription factor bHLH87-like
XM_002280888.1		1618136_at	0.38	0.14	0.16	**4.13**	Ethylene-responsive transcription factor ERF114-like
XM_002274180.2	GSVIVT00033298001	1609559_at	0.00	0.05	0.19	**3.98**	Ethylene-responsive transcription factor ERF003 isoform 1
XM_002272053.1	GSVIVT00003403001		1.02	0.19	0.23	**3.18**	Probable WRKY transcription factor 28
XM_002279376.2	GSVIVT00030359001	1618408_at	0.38	0.09	0.06	**2.89**	Transcription factor bHLH75
XM_002275834.2	GSVIVT00037958001		0.59	0.34	0.19	**2.80**	Ethylene-responsive transcription factor ERF113-like
XM_002285559.1	GSVIVT00015050001		0.15	0.53	0.06	**2.36**	Transcription factor bHLH93
XM_002279450.1	GSVIVT00016545001		0.03	0.04	0.02	**2.32**	Putative transcription factor bHLH041
XM_002284180.2	GSVIVT00025614001		0.07	0.08	0.02	**1.99**	Heat stress transcription factor B-3-like
XM_002264354.2	GSVIVT00007519001		0.17	0.00	0.00	**1.36**	Ethylene-responsive transcription factor ERF098-like
XM_002270623.2	GSVIVT00029005001		0.02	0.04	0.02	**1.34**	Probable WRKY transcription factor 72
XM_002267757.2	GSVIVT00006494001	1607431_at	0.00	0.12	0.00	**1.04**	Probable WRKY transcription factor 53-like
XM_002279303.1	GSVIVT00020055001		0.00	0.00	0.00	**0.96**	Heat stress transcription factor A-6b
XM_003633801.1	GSVIVT00020889001		0.00	0.19	0.17	**0.96**	AP2-like ethylene-responsive transcription factor AIL5-like
XM_002279882.1	GSVIVT00032269001		0.00	0.05	0.00	**0.71**	Transcription factor WER
XM_002277185.2	GSVIVT00020895001		0.05	0.02	0.02	**0.68**	Probable WRKY transcription factor 72
XM_002274351.1	GSVIVT00037881001		0.00	0.00	0.00	**0.62**	Probable WRKY transcription factor 45

Transcriptions factors that are up-regulated 50-fold or greater in E-L 31 berries, and 3-fold or greater in E-L 35-36 berries or E-L 38 berries. The RPKM values indicating specific up-regulation are shown in bold. Expression values are shown in RPKM for each sample, and fold-changes were calculated relative to a minimum value of 0.1. Matching Genoscope and Probeset IDs are shown if applicable and the putative function of encoded proteins are described by their NCBI annotation. A complete list of all differentially regulated transcripts is presented in Additional file 2.

While all of the transcription factors that were 50-fold or greater specifically up-regulated in a single sample were found in young berries, four transcription factors were specifically over-expressed at least 3-fold during veraison, and 29 were specifically over expressed at least 3-fold in ripe berries (Table 4). One veraison-specific transcription factor is of particular interest due to its similarity to, and thus functional annotation as, an UPBEAT1 gene. The UPBEAT1 transcription factor has been shown to control the transition from cell proliferation to cell differentiation in Arabidopsis roots by modifying the balance of reactive oxygen species [45]. In grapes, an oxidative burst has been observed during veraison and is accompanied by the modulation of numerous of ROS scavenging enzymes, including peroxidases, peroxiredoxins, thioredoxins and glutathione-S-transferases [11]. Since many of the transcripts for these enzymes were shown to increase at veraison, the *V. vinifera* UPBEAT1-like transcription factor, XM_003632349.1, could be a potential target for further investigation. In ripe berries, the WRKY family of transcription factors was the most over represented, with 9 out of 58 putative members in grapevine specifically expressed in this sample. WRKY-type transcription factors have previously been implicated in pathogen response pathways in grapes [46,47], and given that berries are most likely to suffer from fungal attack during late ripening stages, the highly regulated expression patterns reported here could be a further indication that some WRKY-type transcription factors are activated in response to biotic stress. Six members of the ethylene-responsive transcription factor family were also up-regulated in ripe berries, although only the ERF113 sub-family was represented by more than a single transcript (Table 4).

### An overview of gene ontology enrichment during berry development

**Figure 3:**
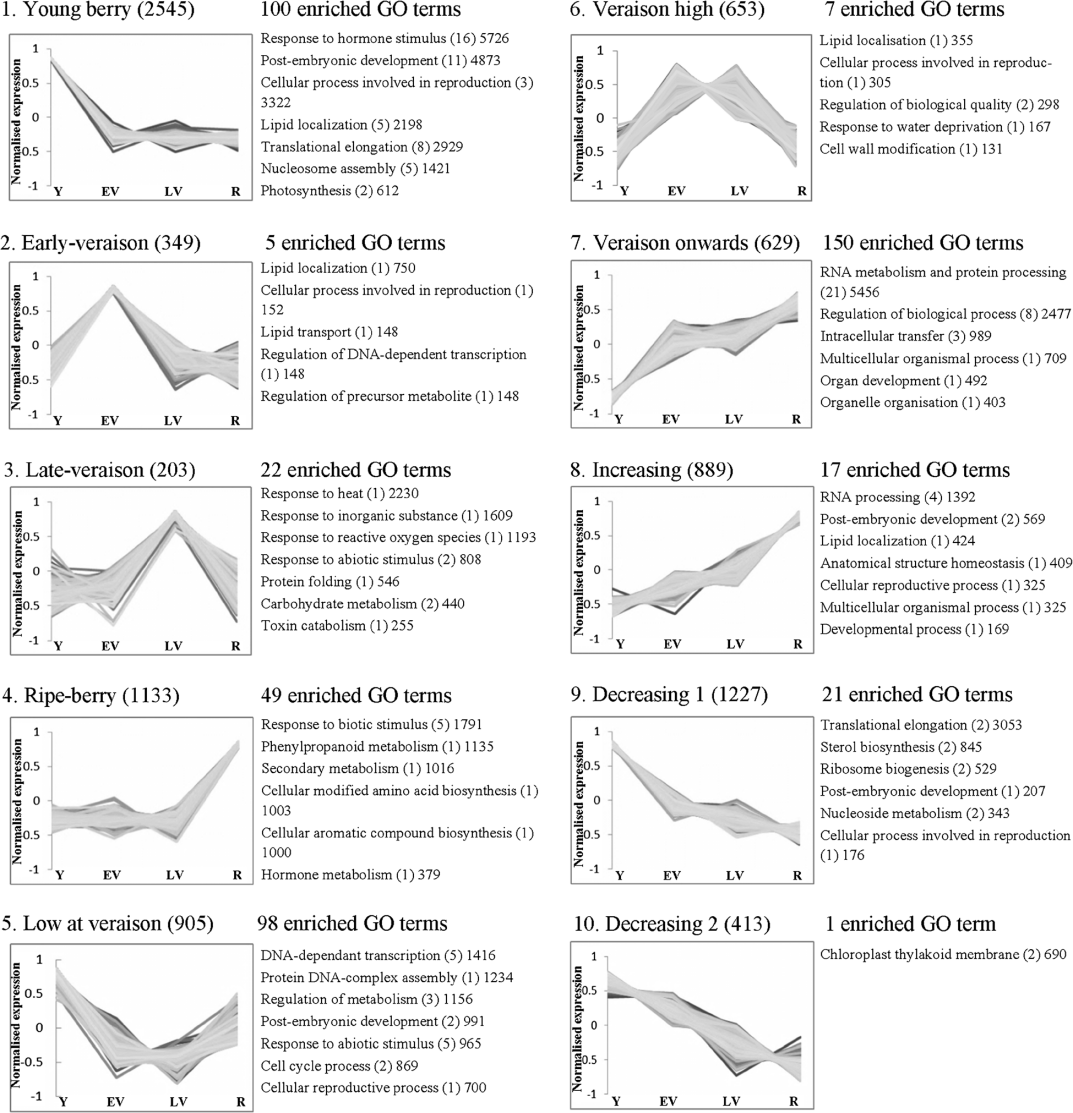
**Clustering and gene ontology enrichment of developmentally regulated transcripts.** Transcripts displaying some degree of developmental regulation were clustered using the K-means method and Euclidean similarity. A description of the pattern of expression and the number of transcripts belonging to the cluster form the title of each chart. Expression values were normalised and scaled between -1.0 and 1.0 (y-axis). Enriched GO terms, generated in AGRIGO and summarised using REVIGO, are listed to the right of each cluster. Only “Biological Process” terms are reported, except for Cluster 10, where the single enriched term was a “Cell Component”. The number of sub-terms combined under the representative description is shown in parentheses, and a value proportional to the statistical significance of enrichment relative to all GO terms in the grapevine transcriptome is given as an indication of the relative level of enrichment (see Methods). Specific transcripts belonging to each presented cluster can be found in Additional file 1: Table S1. Y, young berries (E-L 31); EV, early-veraison (E-L 35); LV, late-veraison (E-L 36); R, ripe berries (E-L 38).

In addition to describing transcripts that were highly up-regulated at a single developmental stage, transcripts that exhibited differential expression between a *number* of time points were investigated using statistical clustering. This technique revealed transcripts from the pool of differentially regulated genes that exhibited similar patterns of expression over the four developmental stages investigated here, regardless of the absolute level of expression. We present 10 clusters of developmentally regulated genes comprising 8,948 transcripts that displayed some degree of differential expression (Figure 3). In agreement with our finding that a large number of transcripts were specifically over expressed in young berries, two of the largest clusters contained transcripts up-regulated in the first developmental stage. Cluster 1 contained 2,545 transcripts that were highly specific to young berries, while cluster 9 contained 1,227 transcripts that were most highly abundant in young berries and exhibited decreasing abundance in later stages. Cluster 10 (413 transcripts) also contained genes that were most abundant in young berries and decreased through to harvest, and cluster 5 (905 transcripts) contained genes that were up-regulated in young and ripe berries, but were less abundant around veraison. The majority of the transcripts reported as specifically up-regulated in young berries based on 3-fold or greater RPKM changes (Table 3 and Additional file 2) fell within clusters 1 and 9. Also consistent with our analysis of stage specific up-regulation presented in Table 3, only 349 and 203 transcripts were specifically up-regulated at either early- or late-veraison, respectively (clusters 2 and 3), while 653 transcripts were up-regulated at both veraison stages (cluster 6). Cluster 4 consisted of 1,133 transcripts that were strongly up-regulated in ripe berries, while cluster 7 (629 transcripts) and cluster 8 (889 transcripts) contained genes for which expression increased throughout development and peaked in ripe berries.

In order to produce a global description of biological processes enriched in each cluster of similarly regulated transcripts, we generated an overview of gene ontology (GO) terms using AgriGO [34]. The AgriGO GO analysis tool retrieved descriptions of gene function based on the standardised vocabulary of the Gene Ontology bioinformatics initiative [48]. We then used the recently-created REVIGO web server to summarise these long lists of GO terms by removing redundant terms and grouping related terms based on semantic similarity [49]. Because GO terms have been assigned using BLAST, Pfam and Interpro scans, individual annotations should be viewed with caution. Nevertheless, for large groups of genes, statistically enriched terms can give insights into biological pathways that are likely to be highly active by comparing them to the frequency at which those GO terms appear in the whole transcriptome. A number of enriched ontological terms were reported several times amongst our clustered transcripts that relate to biological processes which could be expected to be enriched in developing fruit. For example, transcripts annotated with the GO terms “cellular reproductive process” and “post-embryonic development” were found to be enriched in six and five separate gene clusters, respectively (Figure 3). Given that statistical enrichment is calculated in comparison to the whole transcriptome, it is not surprising that GO terms relating to embryo development and reproduction were consistently enriched in berries in general. A more specifically enriched subset of GO terms were those relating to photosynthesis. These were enriched in cluster 1 only, which included transcripts that were highly upregulated in young green berries compered to berries at veraison and harvest, and is in agreement with our initial observation that many transcripts involved in photosynthesis were specifically expressed at this stage. Similarly, GO terms relating to thylakoid membrane localisation were enriched in cluster 10, which consisted of genes that had decreasing abundances throughout development. These results confirm that the gene ontology enrichment detailed here describes biologically relevant metabolic events occurring at different stages of berry development.

An analysis of cluster 4 indicates that secondary metabolic pathways in general were highly up-regulated in ripe berries, as was the biosynthesis of modified amino acids, aromatic compounds, and phenylpropanoids (Figure 3). The most statistically significant enrichment within our cluster analysis was of transcripts involved in responding to hormone signalling, which were highly enriched in cluster 1. This could suggest that overall, hormone-controlled metabolic pathways are most likely to be activated in the early stages of grape development. Additionally, since terms relating to ribosome biogenesis, nucleosome assembly and translational elongation are enriched in clusters 1 and 9, it appears that berries were more translationally active during early development than they were later in the season. This could be explained by the high rate of cell division and differentiation occurring in the weeks following flowering, which later decreases as berry growth increasingly comes about through cell expansion and vacuolar enlargement [1,50]. The GO term “response to heat” was significantly enriched in cluster 3 (late veraison). An in-depth analysis of transcripts located in this cluster revealed that 27 heat shock proteins were present, comprising approximately 13% of the genes in cluster 3, and representing more than one third of all annotated heat shock proteins in *V. vinifera* (Additional file 1). We subsequently found that the minimum temperature on the morning of grape collection at late-veraison was 20.8°C, compared with 12.8-13.7°C on other days of collection and also that the maximum temperature on the day prior to sample collection was the highest of the growing season at 37.8°C (data not shown). Given the well-characterised role of a number of heat shock proteins in response to environmental stimuli such as heat, water stress and oxidative stress [51], this is most likely an example of highly coordinated transcript regulation in response to environmental stimulus, rather than an example of developmental regulation.

A method that has been used previously for the ontological description of grapevine genes is GO-slim, which utilises a simplified subset of GO terms to give a broad overview of ontological content, but assigns many transcripts into vague categories such as “cellular process” or “other biological process” [31]. The descriptive summaries of GO term enrichment generated here using the AgriGO and REVIGO web tools represent a significant advance over previous techniques for ontological description of gene clusters. However, since our analysis of differential transcript expression has been carried out on samples from a specific vineyard over a single growing season, it cannot be inferred that the patterns of transcript expression, and therefore of metabolic pathway activation, are definitively linked with developmental changes. While it is likely that developmentally regulated transcripts have been identified, it is also possible that specific environmental, biotic or abiotic conditions that existed at the time of sampling have played a part in differential transcript regulation. Nevertheless, the differential regulation of transcripts in selected metabolic pathways that was observed during this season will be discussed below, and our full RPKM-based transcript abundance and cluster analyses are detailed in Additional file 1.

### Organic acid metabolism

**Figure 4:**
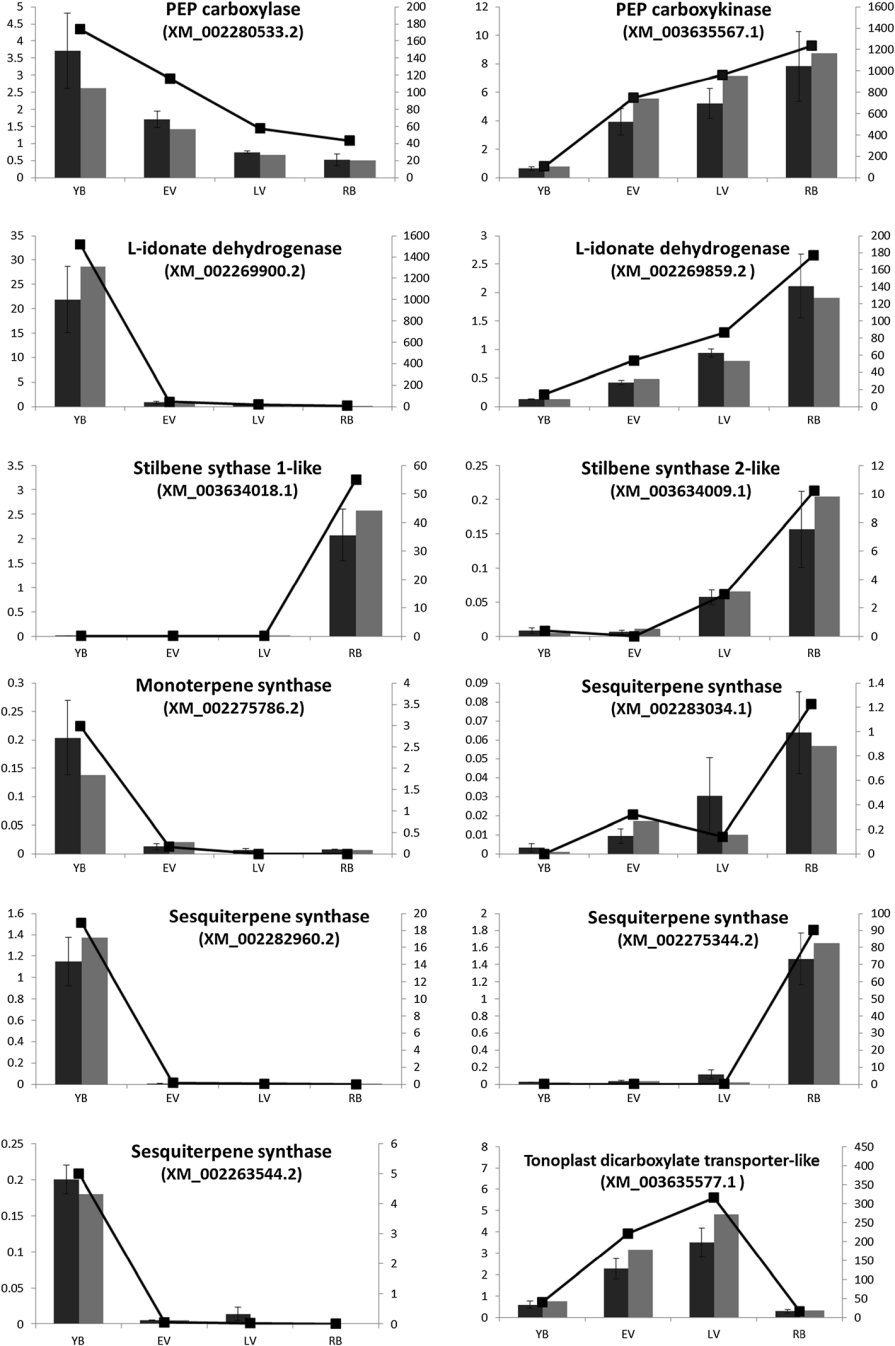
**Quantitative RT-PCR validation of differential transcript expression observed for selected genes.** Comparison of transcript expression for selected genes as measured by RNA-Seq and qRT-PCR. Lines represent expression determined by RNA-Seq in RPKM units (right axis), while histograms represent transcript expression determined by qRT-PCR and normalised to three control genes (left axis; normalised units). Dark grey columns are the average of three biological replicates, with errors bars displaying SEM and light grey columns show the individual replicate on which RNA sequencing was carried out.

The berry metabolism of organic acids including malate, tartrate and ascorbate is an area of active research because of their contribution to juice and wine acidity and to the organoleptic characteristics and ageing potential of wine [3]. Additionally, the malate concentration of harvested berries can affect malolactic acid fermentation and influence the growth of malolactic bacteria [52]. Despite the clear developmentally regulated pattern of malate accumulation and degradation (Figure 1a), the majority of genes encoding enzymes directly involved in malate metabolism, including malate dehydrogenase (MDH) and NAD(P)-dependant malic enzyme, were expressed at all four stages of development investigated, with little differential regulation. Two exceptions to this were isoforms of cytoplasmic MDH (XM_002278600.2) and mitochondrial malic enzyme (XM_002266661.2), which were allocated to cluster 9 and thus decreased through berry development, although transcript abundance remained relatively high (Table 5). Since these two enzymes are involved in malate biosynthesis from oxaloacetate or pyruvate, respectively, their decreasing expression could be reflected in the observed physiological decrease in malate. The constitutive expression of other MDH and malic enzyme isoforms is likely due to the involvement of malic acid in numerous facets of plant primary metabolism, including the tricarboxylic acid cycle and the glyoxylate pathway [3]. In contrast to malate biosynthesis genes, all three transcripts encoding phosphoenolpyruvate carboxykinases (PEPCK; XM_003635567.1, XM_003635619.1 and XM_003635634.1) were allocated to cluster 7 (most highly expressed from veraison onwards), and two transcripts encoding PEP carboxylases (PEPC; XM_002280533.2 and XM_002280806.1) were in cluster 10 (decreasing expression). PEPCK enzymes catalyse the conversion of oxaloacetate to PEP, while PEPC carries out the reverse reaction. Thus, since MDH enzymes catalyse the reversible interconversion of oxaloacetate and malate, the potential decrease in oxaloacetate in mature berries caused by altered expression of PEPC and PEPCK could influence malate degradation by shifting the function of MDH enzymes towards malate catabolism. One isoform each of PEPCK (XM_003635567.1) and PEPC (XM_002280533.2) were included in the qRT-PCR validation of our RNA-Seq analysis, and it was shown that the expression differences observed between the four developmental stages were consistent across three biological replicates (Figure 4). Since the catabolism of malate can only occur when the acid is accessible to metabolic enzymes outside the vacuole, the compartmentation of malate may also influence rates of its accumulation and degradation during berry development. Tonoplast dicarboxylate transporters (TDTs) have been shown to be responsible for the active transport of malate into plant vacuoles [53], and their genomic disruption in Arabidopsis led to decreased malate accumulation [54]. The two transcripts encoding TDTs in grapevine (XM_002277749.1 and XM_003635577.1) were allocated to cluster 7 (highest expression at veraison) and decreased 20-fold between veraison and harvest, and the expression pattern of the latter was confirmed by qRT-PCR (Figure 4). A decrease in malate transport into the vacuole between veraison and harvest, combined with the action of cytoplasmic MDH and PEPCK in malate catabolism, could explain the developmental pattern of malate accumulation and degradation observed in *V. vinifera.*

**Table 5:** Organic acid metabolism

**Encoded protein description**	**Cluster**	**RefSeq accession(s)**
Malate dehydrogenase	9 (decreasing)	XM_002278600.2
	NC	XM_002265044.2, XM_002284873.2, XM_002283583.1, XM_002278676.2, XM_002277507.2, XM_002263634.2, XM_003631644.1, XM_002275406.2, XM_002285320.2
Malic enzyme	9 (decreasing)	XM_002266661.2
	NC	XM_002265729.2, XM_002283715.1, XM_002283778.2, XM_003631725.1
	ND	XM_003631423.1
Phospho*enol*pyruvate carboxylase	10 (decreasing)	XM_002280533.2, XM_002280806.1
	NC	XM_002285405.1
Phosphoenolpyruvate carboxykinase	7 (veraison onwards)	XM_003635567.1, XM_003635619.1, XM_003635634.1
	NC	XM_003632437.1
Tonoplast dicarboxylate transporter	6 (veraison up-regulated)	XM_003635577.1, XM_002277749.1
GDP-Mannose-3,5-epimerase	1 (young berry)	XM_002279341.2
	9 (decreasing)	XM_002283862.2
	10 (decreasing)	XM_003631951.1
GDP-L-galactose phosphorylase (VTC2)	1 (young berry)	XM_002278303.2
	NC	XM_002263621.1
Galactose dehydrogenase	1 (young berry)	XM_002270526.2
L-galactono-1,4-lactone dehydrogenase	NC	XM_002274178.2
L-idonate dehydrogenase	1 (young berry)	XM_002267626.2, XM_002269900.2
	7 (increasing)	XM_002269859.2
Galacturonic acid reductase	5 (low at veraison)	XM_002285191.1
	7 (veraison onwards)	XM_002285183.2

Ascorbate is the main soluble antioxidant in plants and is predominantly synthesised in green tissues by the well-characterised Smirnoff-Wheeler pathway, in which the direct ascorbate precursor L-galactono-1,4-lactone is produced from GDP-L-mannose by the sequential action of GDP-mannose-3,5-epimerase (GME), GDP-L-galactose phosphorylase (VTC2), L-galactose-1-phosphate phosphatase and L-galactose dehydrogenase (L-GalDH) [55,56]. A more recently proposed alternative pathway for ascorbate biosynthesis involves the production of L-galactono-1,4-lactone from D-galacturonic acid by the enzyme galacturonic acid reductase (GalUR) [57]. In a final step, L-galactono-1,4-lactone is converted to ascorbate by L-galactono-1,4-lactone dehydrogenase (GLDH). As a central component of redox metabolism in plants, ascorbate exists in equilibrium with its oxidised form dehydroascorbate, which can be catabolised to oxalate and L-threonate as well as being recycled to ascorbate. The ascorbate catabolic pathway that is of most interest to grape researchers, however, is its conversion into tartrate *via* an L-idonate intermediate; a pathway in which only one enzyme, L-idonate dehydrogenase (L-IdnDH), has been biochemically characterised [58]. In our data, three isoforms of GME (XM_002279341.2, XM_002283862.2 and XM_003631951.1) were allocated to clusters 1, 9 and 10, indicating that they were specifically expressed in young berries, or were most abundant in young berries and then decreased during ripening (Table 5). Similarly, the single isoform of L-GalDH (XM_002270526.2) and the most abundant isoform of VTC2 (XM_002278303.2) were allocated to cluster 1. Also, although GLDH (XM_002274178.2) was not differentially expressed enough to be allocated a cluster in our analysis, its abundance did decrease during development and was 3-fold lower in ripe berries than in immature berries (Additional file 1). Given that both ascorbate and tartrate levels have been shown to increase in grape berries most rapidly from about two weeks after flowering until veraison [56], our data suggests that this is potentially controlled transcriptionally through differential expression of components of the Smirnoff-Wheeler pathway. Comparable results were obtained for genes of this pathway investigated with quantitative real-time polymerase chain reaction and reported by Melino *et al.* (2009). The transcript most similar to the characterised *GalUR* from strawberry (XM_002285191.1) was detected at very low levels in young berries, and not at all in the other samples. However, several other transcripts encoding putative oxidoreductases that are also homologues of *GalUR* were expressed at much higher levels, including XM_002285183.2, which was allocated to cluster 7 (veraison onwards). Two of the three potential L-IdnDH isoforms were specific to young berries and located in cluster 1 (XM_002267626.2 and XM_002269900.2) while a third isoform (XM_002269859.2) was in cluster 7, suggesting that the biosynthesis of tartrate from ascorbate may be controlled at different stages of grape development by different genes (Table 5). The transcript expression levels for two L-IdnDH isofroms, XM_002269900.2 and XM_002269859.2, were validated by qRT-PCR, and demonstrated that the patterns were consistent across three replicates from different vines (Figure 4).

### Co-regulation of phenylpropanoid/stilbene biosynthetic genes

A grape secondary metabolite that has received a great deal of attention in recent times is the polyphenolic compound resveratrol (3,5,4’-trihydroxy-trans-stilbene). Resveratrol is a phytoalexin involved in pathogen defence in grapevine [59], although it has also been shown to be present in healthy grapes [60]. Resveratrol is found in red wine and can positively regulate a number of beneficial physiological processes in animals [61]. The resveratrol biosynthesis pathway consists of four enzymes that sequentially transform phenylalanine into this specialised secondary metabolite. The first three enzymes, phenylalanine ammonia lyase (PAL), cinnamic acid 4-hydroxylase (C4H) and 4-coumarate:CoA ligase (4CL), are components of the common phenylpropanoid pathway, which also leads to the production of phenolic compounds such as lignins, anthocyanins and other flavonoids. The fourth enzyme, stilbene synthase (STS), exists only in plants that produce stilbenes, and can catalyse the final step by converting 4-coumaroyl-CoA and three molecules of malonyl-CoA into *cis-* or *trans-*resveratrol. Although this biosynthetic pathway is commonly described as comprising four single enzymes, each are encoded by multi-gene families, which have potentially redundant activities, and may have different temporal or spatial expression. The NCBI annotation of proteins encoded by the RefSeq mRNA transcripts suggests that there are 12 PALs, 3 C4Hs, 12 4CLs and a startling 38 STSs. Despite the fact that less than 35% of the RefSeq mRNAs were differentially expressed enough to be included in our cluster analysis, almost all the transcripts in the stilbene biosynthesis pathway were assigned to a cluster. This confirms the well-reported observation that both general phenylpropanoid metabolism and specialised resveratrol metabolism are highly regulated processes in grapes. The majority of PAL, C4H and STS transcripts were grouped in cluster 4, indicating they were specifically up-regulated in ripe berries. In contrast, the 4CL transcripts exhibited more varied expression patterns, including three transcripts in cluster 1 (young fruit), two in cluster 5 (low at veraison) and one each in clusters 8 (increasing), 9 and 10 (decreasing; Table 6). A PAL transcript has previously been reported to be up-regulated early in the season under water-deficit, as measured by the intensity of the GeneChip probeset 1613113_at (corresponding to XM_0022272890.1) [62]. This particular transcript was the only one of 12 putative grapevine PAL genes which was not assigned a cluster in our analysis, and therefore the specific co-regulation of the majority of PAL genes in ripe berries that we saw in our data was not observed in that study. Guillaumie *et al.* (2011) reported that two PAL isoforms, corresponding to XM_002281763 and XM_002267917, increased in abundance over the final week of ripening, which is consistent with our results for these transcripts [13]. The first three steps of the phenylpropanoid pathway provide 4-coumaroyl CoA as a substrate for chalcone synthase (CHS), which produces chalcone as the precursor for the important organoleptic flavonoids and anthocyanins. While seven potential CHS transcripts are annotated in the RefSeq mRNA collection, three of these were not detected in our data and may represent *V. vinifera* genes expressed in tissue other than grape. Two of the four detectable CHS transcripts were grouped in cluster 1 (XM_002276885.2 and XM_002276910.1), one was in cluster 5 (XM_002263983.1), and one was not differentially regulated (XM_002276617.1).

**Table 6:** Phenylpropanoid/stilbene pathway transcripts

**Encoded protein description**	**Cluster**	**RefSeq accession(s)**
Phenylalanine ammonia-lyase	1 (young berry)	XM_002285241.1
	2 (early veraison)	XM_002278480.2
	4 (ripe berry)	XM_002268220.2, XM_002267917.2, XM_003633939.1, XM_002268145.2, XM_003633937.1, XM_003633938.1, XM_002268737.2, XM_002268696.2
	5 (low at veraison)	XM_002281763.2
	NC	XM_002272890.1
Cinnamic acid 4-hydroxylase	4 (ripe berry)	XM_002266106.1, XM_002266001.1
	5 (low at veraison)	XM_002266202.1
4-coumarate:CoA ligase	1 (young berry)	XM_002285884.2, XM_002285885.1, XM_002274958.2
	5 (low at veraison)	XM_002265509.1, XM_002272746.2
	8 (increasing)	XM_002270324.1
	9 (decreasing)	XM_002279486.2, XM_002270556.1
	NC	XM_002276317.2
	ND	XM_002271550.2, XM_002269909.1, XM_002266436.2
Stilbene synthase 1-like	4 (ripe berry)	XM_002264419.2, XM_002263926.1**a**, XM_002263845.2, XM_003634014.1**a**, XM_002263686.2, XM_003634018.1, XM_003634015.1, XM_003634017.1
Stilbene synthase 2-like	4 (ripe berry)	XM_002265955.1, XM_002278447.2, XM_002278349.1, XM_002265193.2, XM_002271335.2, XM_002268806.2**b**, XM_003634020.1**b**, XM_002272093.2, XM_003634032.1
	8 (increasing)	XM_003634009.1
Stilbene synthase 4-like	4 (ripe berry)	XM_002264953.2, XM_002278263.2, XM_002269257.2, XM_003634025.1, XM_003634026.1, XM_003634021.1, XM_003634022.1, XM_003634019.1, XM_003634028.1, XM_003634023.1, XM_003634024.1, XM_003634027.1
Stilbene synthase 5-like	4 (ripe berry)	XM_002268720.2, XM_002278318.2, XM_002263999.2, XM_002269350.2,
	ND	XM_002263927.1
Stilbene synthase 6-like	4 (ripe berry)	XM_002262908.2, XM_002263771.2, XM_003634016.1
Chalcone synthase	1 (young berry)	XM_002276885.2, XM_002276910.1
	2 (low at veraison)	XM_002263983.1
	NC	XM_002276617.1
	ND	XM_002276606.1, XM_002269415.2, XM_003634008.1

Clustering of genes involved in phenylpropanoid metabolism and stilbene biosynthesis. NC, expressed but not clustered; ND, not detected; **a** transcripts with the highest sequence similarity to the functionally characterised resveratrol synthase, Vst1 [65]; **b** transcripts with the highest sequence similarity to the functionally characterised resveratrol synthase, StSy [64].

Several *V. vinifera* STSs have been shown biochemically to be involved in resveratrol biosynthesis [63-65], however the high sequence similarity amongst this multi-gene family (85-99% identity) suggests they may all carry out a similar, or identical, biochemical reaction. Thus, an accurate description of the expression of each isoform is required for a full understanding of the conditions and tissue in which resveratrol is likely to be produced. Our data indicated that 36 of the 38 STSs were co-regulated in cluster 4, one was not detected, and one was in cluster 8, which also consisted of genes most highly expressed at harvest (Table 6). A high proportion of reads mapped to each RefSeq STS transcript were unique, even when RPKM counting was performed at 99% (data not shown), suggesting that the strong co-regulation of this gene family was not an artefact of the read mapping process. Eight STSs are represented by probesets on the GeneChip microarray platform, and DeLuc *et al.* (2011) demonstrated that each of these was up-regulated to some degree late in the growing season, with highest expression from five weeks after veraison until harvest [66]. They also demonstrated that this up-regulation was increased in water deficit conditions, so it is possible that the environmental conditions during the season under investigation here could have contributed to, or been the cause of, the highly coordinated up-regulation of STSs in ripe berries. A more recent investigation into stilbene synthase expression during grape ripening with the latest and most comprehensive microarray platform showed low expression of STSs in all stages of berry development until post-harvest [67], suggesting that the precise timing of berry harvest could be a vital determinant in stilbene, and thus resveratrol, content in wine. We investigated the expression levels to two STSs across our four developmental stages *via* quantitative RT-PCR, including the single STS that was located in cluster 8 (XM_003634009.1), and one of the STSs located in cluster 4 due to its specific expression in ripe berries (XM_003634018.1). This PCR-based method validated the result observed from our RNA-Seq data, and demonstrated that the results were consistent amongst the three biological replicates analysed (Figure 4).

### Differential expression of aroma-related transcripts

Aroma is an important determinant of wine quality, and the precursors of many aroma compounds found in wine are synthesised during berry development. Compounds from the terpenoid class of biochemicals have been shown to influence the aroma of wine, with several 10-carbon monoterpenes affecting the fruity character of wine [68], and a 15-carbon sesquiterpenoid being responsible for the peppery aroma of Shiraz [69,70]. Monoterpenes are formed through the action of terpene synthase-a (TPS-a; [29]) enzymes that use geranyl pyrophosphate as a substrate, arising from products of the deoxy xylulose-5-phosphate (DXP) pathway, isopentenyl pyrophosphate (IPP) and dimethylallyl pyrophosphate (DMAPP). The DXP pathway consists of seven chloroplast-localised enzymes [71], for which six of the encoding transcripts were expressed at all four stages of berry development with little differential regulation. The transcript encoding the final enzyme of the DXP pathway, hydroxymethylbutenyl diphosphate reductase (XM_002284623.2) was in cluster 7 and therefore up-regulated at veraison and in ripe berries (Table 7). Although transcripts encoding components of the DXP pathway were expressed during berry development, we detected almost no expression of putative monoterpene synthases. Similar to the number of putative TPS-a genes identified by Martin *et al.* (2010) [29], 29 potential monoterpene synthases were found in the RefSeq mRNA collection, all of which were annotated by sequence similarity as myrcene or linalool synthases. Of these 29 transcripts, 26 were not detected in any of the four developing grape samples investigated here. The other three were detected at relatively low transcript abundances (RPKM < 5), with one each in clusters 1 and 10, and one expressed in the first two stages but not assigned to a cluster (XM_002275786.2, XM_002276009.1 and XM_003634850.1, respectively). The specific expression of XM_002275786.2 in immature green berries when compared with berries at veraison or harvest was confirmed by quantitative RT-PCR (Figure 4). Given the high transcriptome coverage observed in each sample and therefore our ability to detect transcript expression at extremely low levels, this is a strong indication that TPS-a enzymes do not play an important metabolic role for *V. vinifera* (cv. Shiraz) during ripening. In contrast to the absence of expression of monoterpene synthases in ripening Shiraz berries, the expression of a linalool/nerolidol synthase was recently found to be highest during veraison in the Gewürztraminer grape variety [72]. Additionally, although significant levels of monoterpenes such as geraniol, linalool and α-terpineol are found in Muscat grapes [73] and to a lesser extent in Gewürztraminer and Riesling varieties [72,74], they have not been found at significant levels in red grape varieties. The low level of expression of three putative monoterpene synthases in the earliest Shiraz berry sample (E-L 31) could be a reflection of transcriptional events that were up-regulated during flowering, when monoterpene synthases have been shown to be transcribed [75]. In the absence of monoterpene synthase expression in ripening berries, the presence of transcripts encoding the DXP pathway can be explained by the potential utilisation of IPP and DMAPP for the biosynthesis of other terpene-based metabolites such as carotenoids and phytosterols.

**Table 7:** Terpenoid pathway transcripts

**Encoded protein description**	**Cluster**	**RefSeq accession(s)**
4-hydroxy-3-methylbut-2-enyl diphosphate reductase	7 (veraison onwards)	XM_002284623.2
TPS-a (monoterpene synthases)	1 (young berry)	XM_002275786.2
	2 (decreasing)	XM_002276009.1
	NC	XM_003634850.1
	ND	XM_003633271.1, XM_003635303.1, XM_002265375.2, XM_003633272.1, XM_003634832.1, XM_003634833.1, XM_002275070.1, XM_003634834.1, XM_003634838.1, XM_002267425.2, XM_002267123.1, XM_003634855.1, XM_002274758.2, XM_003635585.1, XM_003634831.1, XM_002267417.1, XM_003634835.1, XM_003634836.1, XM_003634837.1, XM_003634854.1, XM_002266772.1, XM_002266983.2, XM_002275237.1, XM_002279833.2, XM_003635411.1, XM_003635502.1
Hydroxymethylglutaryl-coenzyme A reductase	9 (decreasing)	XM_002275791.2, XM_002265602.1
	NC	XM_002283147.2
Farnesyl pyrophosphate synthase	9 (decreasing)	XM_002272605.2
TPS-b (sesquiterpene synthase)	1 (young berry)	XM_003634648.1, XM_002282960.2, XM_002263544.2
	2 (early veraison)	XM_002282452.1
	4 (ripe berry)	XM_002275344.2, XM_002274745.2, XM_002274409.2, XM_002275372.2, XM_002283034.1
	6 (veraison up-regulated)	XM_002276330.2
	ND	XM_003634900.1, XM_003634901.1, XM_002275315.1, XM_002277227.2, XM_002273588.2, XM_002277315.2, XM_002275101.2, XM_002275554.2, XM_002275022.1, XM_002285472.1, XM_002283040.2, XM_002283308.1, XM_003634597.1
Carotenoid cleavage dioxygenase	4 (ripe berry)	XM_002268368.2
	7 (veraison onwards)	XM_002278714.2, XM_002278592.2, XM_002270125.1
	ND	XM_002281203.1, XM_002274162.1, XM_002269502.2, XM_002281297.2, XM_003631732.1, XM_003633051.1

Sesquiterpenes are produced by members of the TPS-b enzyme family from farnesyl pyrophosphate (FPP), which is formed in the cytoplasm from IPP and DMAPP. Cytoplasmic IPP and DMAPP are produced by the mevalonate pathway, consisting of six enzymes for which transcripts were found in each of the four developmental stages. From the mevalonate pathway, two of the three transcripts encoding isoforms of hydroxymethylglutaryl-coenzyme A reductase (HMGR) were in cluster 9 (decreasing expression through development), as was FPP synthase, while all other transcripts were unclustered. HMGR is considered to be the rate limiting enzyme in the mevalonate pathway [76], and thus its up-regulation early in development could indicate a greater requirement for terpene precursors in immature berries. We identified 23 transcripts encoding putative TPS-b enzymes, which are currently annotated by NCBI as valencene synthase-like or germacrene synthase-like genes. The differential regulation of TPS-a and TPS-b transcripts in grapes has not previously been reported in detail in microarray experiments due to poor coverage of the TPS gene family by the available probes. For example, on the Affymetrix GeneChip there is only a single probe that interrogates a TPS-a transcript and four probes that interrogate TPS-b transcripts (Additional file 1). In our analysis, however, 10 of the 23 TPS-b transcripts were detected in at least one sample, and all 10 exhibited differential expression during grape development (Table 7). Three transcripts were in cluster 1, and were therefore specifically expressed in young berries (XM_003634648.1, XM_002282960.2 and XM_002263544.2), two were specifically expressed around veraison and allocated to clusters 2 and 6 (XM_002282452.1 and XM_002276330.2, respectively), and five transcripts were in cluster 4 and up-regulated in ripe berries (XM_002275344.2, XM_002274745.2, XM_002274409.2, XM_002275372.2 and XM_002283034.1). Remarkably, all of these transcripts except XM_002276330.2 were predominantly expressed in only one of the four samples, demonstrating the existence of tightly controlled differential regulation. Given the importance of some sesquiterpenoids for the aroma of wine (e.g. [69]), members of the TPS-b clade of terpene synthases for which transcripts are up-regulated in ripening berries may be interesting future targets for functional characterisation. We validated the observed differential expression for two transcripts from cluster 1 and two from cluster 4 using qRT-PCR. For three of these transcripts, we confirmed that the extremely specific temporal expression was consistent amongst three biological samples, while in the case of transcript XM_002283034.1, it was relatively highly expressed at the late-veraison stage as well as ripe berries, in one of the three biological replicates (Figure 4).

Another class of potential aroma compounds that stem from terpene metabolic pathways are the C13 norisoprenoids, such as β-damascenone and ionone, which are derived as breakdown products of C20 carotenoids [77]. The breakdown of carotenoids into norisoprenoids is thought to be catalysed by carotenoid cleavage dioxygenase (CCD) enzymes, one of which has been functionally characterised in grapes (VvCCD1) [78]. The transcript encoding VvCCD1, XM_002278714.2, was grouped in cluster 7, and was highly abundant (RPKM > 200) from early-veraison through ripening, while a close homologue XM_002278592.2 was expressed at much lower level but followed a similar expression pattern (Table 7). Transcripts encoding two other putative CCDs were detected in our samples, including XM_002270125.1, which was also grouped in cluster 7, and XM_002268368.2, which was in cluster 4 and highly up-regulated in ripe berries. This last observation is in agreement with a recent microarray study by Guillaumie *et al.* (2011), who reported that XM_002268368.2 expression increased approximately 2-fold in the final week of ripening [13]. Our data therefore provides an indication that the production of C13 norisoprenoids by the CCD-catalysed enzymatic cleavage of carotenoids is initiated at veraison and continues through until harvest, and could explain the physiological observation that β-damascenone accumulates after veraison [79].

## Conclusions

RNA-Seq analysis of transcript abundances during berry development has enabled us to carry out a global investigation of gene expression at four time-points in developing grapes and has facilitated a comprehensive description of differential transcriptional events that occurred within a single season for the important wine grape variety *V. vinifera* (cv. Shiraz). We have reported a detailed description of the expression profiles of 23,720 mRNA transcripts contained within the NCBI RefSeq *V. vinifera* collection, and shown that this is an accurate reference for transcript abundance measurements. We used gene clustering and the enrichment of Gene Ontology terms to describe the overall biological processes that were regulated during development, and described the transcriptional patterns of genes involved in organic acid, stilbene and terpenoid metabolism as examples of co-regulated and differentially expressed gene families. Quantitative real-time PCR was used to confirm the differential expression patterns observed for 12 of the genes reported, and it was demonstrated that the results obtained with RNA-Seq were consistent with the average expression from three biological replicates. Whether the differential regulation of gene expression described here occurred solely as a consequence of berry development, or in response to specific environmental, biotic or abiotic conditions requires further confirmation during other seasons and in different locations. Also, the extent to which the differential regulation of genes reported here is applicable to other *V. vinifera* varieties is yet to be shown, and the investigation of transcriptional changes at more closely spaced developmental stages will provide further valuable information. Our full transcript abundance analysis, presented in Additional file 1, represents an invaluable resource for hypothesis development and candidate gene selection.

## Methods

### Sample collection and berry developmental measurements

*V. vinifera* (cv. Shiraz) bunches of vines grown at the Nuriootpa Research vineyard, Barossa Valley, South Australia, were tagged at 50% cap-fall. Three replicates of 20 berries were harvested throughout the 2010-11 season between 9-10 am. Individual replicates consisted of berries from different vines, and each replicate consisted of two berries taken from random positions on each of ten bunches on that vine. Harvesting was carried out by cutting through the pedicel at the junction between stem and berry, frozen immediately in liquid nitrogen, and subsequently stored at -80°C until required. For the purposes of total soluble solids (TSS) estimation, additional fruit (12 berries per sample) was collected and individual berries analysed for °Bx with a digital Pocket Refractometer (Atago, Tokyo). Developmental stages were characterised by changes in berry weight accumulation, TSS and malic and tartaric acid concentration as well as observed changes in berry colour and deformability.

For determination of malic acid and tartaric acid content, each replicate of 20 frozen whole berries was ground to a fine powder in a liquid nitrogen-cooled A11 basic mill (IKA, Germany). Organic acids were extracted from 0.3 g of powder in 0.5 M *ortho*-phosphoric acid, pH 1.5, in a final volume of 1.5 ml. Samples were mixed for 1 hour at room temperature and centrifuged at 16,000 g for 10 mins. The supernatant was passed through a 45 μm PVDF 30 mm filter and malic and tartaric acids were quantified using reversed phase HPLC on an Agilent 1100 series HPLC (Agilent Technologies, Santa Clara, USA). The extract (20 μl) was injected into a Kinetex™ 2.6 μm C18 100Å column (150 mm × 4.6 mm ID) with guard column (Phenomenex, Sydney, Australia), maintained at 30°C. The mobile phase was 10 mM KH_2_PO_4_ (pH 2.9) with a flow rate of 0.5 ml/min. Detection was carried out at 210 nm with a photodiode array detector, and concentrations were determined according to calibration curves of appropriate standards using Chemstation for LC 3D systems software (Agilent Technologies, Santa Clara, USA).

### RNA extraction and sequencing

For large scale RNA extraction for next generation sequencing, approximately 2 g of powder from one of the replicates of harvested berries was ground further with a mortar and pestle and added to 15 ml RNA extraction buffer [80], pre-warmed to 65°C, consisting of 2% (w/v) cetyltrimethylammonium (CTAB), 2% (w/v) polyvinylpyrrolidone (PVP) K-20, 100 mM TRIS-HCL (pH 8.0), 25 mM EDTA, 2.0 M NaCl, 0.5 g l^-1^ spermidine, with 2% (v/v) 2-mercaptoethanol added immediately prior to use. Samples were mixed by vortexing and incubated at 65°C for 10 min with gentle mixing every 3 min, prior to the addition of 10 ml 24:1 chloroform-isoamylalcohol (CIA). Samples were centrifuged at 10 000g for 10 min at room temperature, and the top aqueous layer was transferred to fresh tubes. Washes were repeated twice with 10 ml CIA and 4 ml 10 M LiCl was added to the final aqueous layer, which was incubated overnight at 4°C. Samples were centrifuged at 10 000 g for 30 min at 4°C, the supernatant was removed and the pellet was resuspended in 800 μl STE buffer containing 1M NaCl, 10 mM TRIS pH 8.0 and 1 mM EDTA pre-heated to 65°C. The resuspended RNA was washed once with 800 μl CIA and the aqueous layer transferred to a fresh tube. For a final RNA precipitation, 300 μl cold isopropanol and 300 μl of salt solution containing 1.2 M sodium citrate and 0.8 M NaCl were added, samples were incubated at -20°C for 10 min, and centrifuged at 10 000g for 20 min at 4°C. The supernatant was discarded, and 1 ml cold ethanol was added to the pellet. Samples were centrifuged once more at 10 000g for 10 min, and the supernatant was removed. The pellet was air dried, and resuspended in 100 μl DEPC (diethylpyrocarbonate) treated water. RNA integrity and concentration were determined using a Nanodrop 2000 (Thermo Scientific), and samples were diluted to approximately 300 ng μl^-1^ in TE buffer. RNA for qRT-PCR analysis of the other two biological replicates was extracted by the same method, except 200 mg of ground grape tissue was used and volumes were adjusted accordingly.

Illumina RNA sequencing was carried out at the Australian Genome Research Facility (AGRF, Melbourne, Australia) on an Illumina HiSeq 2000 instrument (Illumina). RNA quality control was carried out on a 2100 Bioanalyzer (Agilent Technologies) and each sample received an RNA integrity numbers (RIN). Poly(A) mRNA was prepared and sequences from each of the four developmental stages were indexed with unique nucleic acid identifiers. Sequencing on the HiSeq 2000 was carried out according AGRF protocols and following the manufacturer’s instructions for the generation of single-end reads, and data was generated with CASAVA 1.8.1 pipeline (Illumina). The sequence reads from all four samples were analysed according to AGRF quality control measures; adaptor or contaminant sequences were removed and reads containing long stretches of ambiguous characters were clipped.

### Sequencing data analysis

For mapping sequence reads against the most recently curated non-redundant mRNA transcriptome, 23720 sequences of *V. vinifera* RefSeq mRNAs [33] were retrieved from the National Centre for Biotechnology Information (http://ncbi.nlm.nih.gov) and CLC Genomic Workbench 4.8 (CLC Bio) was used to assemble the cleaned sequence data against this single reference file in FASTA format. Prior to transcriptome mapping, two nucleotides were trimmed from each end of each sequence read, and reads under 60 nucleotides in length or with greater than two ambiguous nucleotides were not included in the mapping or counting. For inclusion in the calculation of RPKM values, cut-offs were set such that greater than 50% of a read in contiguous nucleotides must have aligned to a reference transcript with greater than 98% identity. When reads could be mapped to multiple reference locations, they were assigned to reference transcripts proportionally based on the relative number of unique reads already mapped to each of the reference sequences.

### Quantitative real-time polymerase chain reaction

Quantitative RT-PCR was carried out on cDNA generated from three biological replicates harvested as described above, one of which corresponded to the sample subjected to Illumina sequencing for RNA-Seq analysis. Reactions were set up in KAPA SYBR Fast qPCR Universal ReadyMix (Geneworks, Adelaide, Australia) according to manufacturer’s instructions, with gene-specific primers (0.125 μM) in a final volume of 20 μl. Details on gene annotations, accessions and primer sets are included in Additional file 3. Thermal cycling conditions involved an initial 95°C melt (3 min), followed by 40 cycles of 95°C (3 s) and 60°C (30 s). Assays were conducted with a C1000 Thermal Cycler fitted with a CFX96 Real-time PCR detection system (BioRad), and analysed using the CFX Manager software (BioRad). Primer pairs were designed to target unique regions of the genes of interest, and PCR and agarose gel analyses were used to verify the absence of non-specific amplification prior to qRT-PCR. Additionally, following reactions DNA melt curves were created for each primer combination to confirm the presence of a single product. The average of two technical repeats was used for each reaction, and the standard error of the mean was calculated for the three biological replicates. Transcripts were normalized to a reference number derived from transcript levels of three reference genes; namely ankyrin-repeat domain protein, elongation initiation factor (eIF-2B) and calcineurin B-like protein.

### Global comparison of transcript expression between technical platforms

Relevant publically available grape Affymetrix probesets were retrieved from http://www.affymetrix.com and microarray raw data were downloaded from PlexDB [81]. Raw CEL files were processed using RMAExpress software (http://rmaexpress.bmbolstad.com) using the background-adjusted and quantile normalised setting, and intensity data was summarised using robust multiarray average (RMA) expression values. Cross-hybridizing probesets (represented by the _s_, _x_ and _a_ identifiers) were removed and BLASTn analysis of the express sequence tags (ESTs) on which the remaining probesets were designed was conducted against the current NCBI RefSeq *V. vinifera* mRNA dataset using PERL. Only probesets for which the ESTs matched a RefSeq transcript with an e-value of zero were considered, and where more than one probeset matched to a single RefSeq transcript only the most closely correlating probeset was included. The signal intensities for probesets fulfilling the above requirements were log2 transformed and equivalent expression values from RNA-seq were obtained by calculating log2 (RPKM + 1) to avoid taking the log of zero. Spearman correlation coefficients between global relative expression and individual transcript expression patterns were calculated using Microsoft Excel for each developmental stage, and an average of the four stages is presented. While Pearson product-moment correlation coefficients yielded similar results, Spearman coefficients were reported due to the non-linear relationship between microarray intensity and absolute expression [82]. When removing transcripts that were not considered expressed from our datasets, probesets with intensities below the 25^th^ percentile (corresponding to a normalised intensity of 4.0) and transcripts with an RPKM < 0.5 were discarded.

### Cluster analysis and gene ontology assignment

Clustering of transcript expression patterns based on NCBI RefSeq RPKM levels was carried out with Cluster 3.0 [83]. Prior to cluster formation, transcripts that had an RPKM value below 0.5 in each stage were discarded. RPKM expression values for each transcript were normalised to between -1.0 and 1.0 by multiplying by a scale factor such that the sum of the squares of the four values for each transcript was 1.0. The normalised expression values for each transcript were then centred on zero by subtracting the mean of the four values from each data point so that the mean of each row was zero. Transcripts that displayed a difference of less than 0.5 between the maximum and minimum normalised data points were filtered to select for genes displaying a significant degree of differential regulation. Clustering was carried out using the k-means method for 20 clusters and with the Euclidean similarity metric. After 1000 iterations the reported clustering result was found three times (details can be found in the Cluster 3.0 manual; [83]). RefSeq accessions were compiled from each cluster and their corresponding Genoscope (8x assembly) accession, if available, were input into the AgriGO agricultural gene ontology (GO) analysis tool (http://bioinfo.cau.edu.cn/agriGO/analysis.php) to elucidate enriched GO terms within the cluster when compared with GO terms in the complete *V. vinifera* transcriptome [34]. The REVIGO web server (http://revigo.irb.hr/) was used to summarise the biological processes represented in the lists of significantly enriched GO terms from each cluster [49]. Only Biological Process GO terms with a false discovery rate (FDR; e-value corrected for list size) of ≤ 0.05 were submitted to the REVIGO tool, and the “small allowed similarity” setting was selected to obtain a compact output of enriched biological processes. The overall significance of enriched biological processes was expressed as the sum of 100 x -log_10_(FDR) for each enriched GO term counted within that process, giving an arbitrary value proportional to the relative statistical significance at which the biological process was enriched. For example, using this technique a biological process including a single enriched term with a FDR of 0.01 would give a value of 200, while an FDR of 1x10^-10^ would give 1000. This technique was adapted from the method used to visualise enriched GO terms as a percentage of the total enriched terms in the TreeMap function of the REVIGO web server [49].

## Competing interests

The authors declare that they have no competing interests.

## Authors’ contributions

CS coordinated sample collection and physiological analysis, participated in transcriptomic data analysis and carried out the qRT-PCR. DW carried out the computational comparison and statistical analysis of RNA-Seq and microarray data. CMF participated in the design and coordination of the study and assisted in drafting the manuscript. DPD conceived the study, isolated berry RNA, compiled and analysed transcriptome sequencing data and drafted the manuscript. All authors read and approved the final manuscript.

## Supplementary Material

Additional file 1 **Absolute expression levels for all NCBI RefSeq transcripts in stages EL 31, 35, 36 and 38 of developing Shiraz grape.** Additional file 1 consists of three tables in Microsoft Excel format. Table 1 contains RPKM data for all NCBI RefSeq *Vitis vinifera* transcripts at developmental stages E-L 31, E-L 35, E-L 36 and E-L 38, and includes their NCBI putative functional annotation, and the closest matching Genoscope accession and Affymetrix Probeset ID for the purpose of cross-referencing to other work. Each Probeset ID is listed once only, and matches to its most similar RefSeq transcript. The cluster to which each gene has been allocated with regards to Figure 3 is also shown. Table 2 contains transcripts expressed with an RPKM of greater than 200 at all four stages of berry development under investigation. Corresponding Genoscope annotations and Affymetrix GeneChip probeset IDs have been assigned through BLASTx and VitisNet Network or Category functional annotations are taken from [32]. Table 3 contains a complete list of all RefSeq transcripts not detected with at least five unique sequencing reads, or with and RPKM ≥ 0.5 in any of the samples. Click here for file

Additional file 2 **List of all NCBI RefSeq transcripts specifically up-regulated 3-fold or greater at a single developmental stage, or during veraison.** Additional file 2 consists of a Microsoft Excel File containing five worksheets, containing lists of transcripts specifically up-regulated at each developmental stage, or during both veraison stages. RPKM data are also included, as is the NCBI putative functional annotation. Click here for file

Additional file 3 Primers used for qRT-PCR analysis presented in tabulated format. Microsoft Excel File containing a single worksheet with primer sequences shown for each of 15 genes, including 3 control genes, alongside the gene annotation and accession number. Click here for file
